# Transcriptomic Profiling of Zebrafish Hair Cells Using RiboTag

**DOI:** 10.3389/fcell.2018.00047

**Published:** 2018-05-01

**Authors:** Maggie S. Matern, Alisha Beirl, Yoko Ogawa, Yang Song, Nikhil Paladugu, Katie S. Kindt, Ronna Hertzano

**Affiliations:** ^1^Department of Otorhinolaryngology Head and Neck Surgery, University of Maryland School of Medicine, Baltimore, MD, United States; ^2^Section on Sensory Cell Development and Function, National Institute on Deafness and Other Communication Disorders, Bethesda, MD, United States; ^3^Institute for Genome Sciences, University of Maryland School of Medicine, Baltimore, MD, United States; ^4^Department of Anatomy and Neurobiology, University of Maryland School of Medicine, Baltimore, MD, United States

**Keywords:** inner ear, hair cells, zebrafish, RiboTag, RNA-Seq

## Abstract

The zebrafish inner ear organs and lateral line neuromasts are comprised of a variety of cell types, including mechanosensitive hair cells. Zebrafish hair cells are evolutionarily homologous to mammalian hair cells, and have been particularly useful for studying normal hair cell development and function. However, the relative scarcity of hair cells within these complex organs, as well as the difficulty of fine dissection at early developmental time points, makes hair cell-specific gene expression profiling technically challenging. Cell sorting methods, as well as single-cell RNA-Seq, have proved to be very informative in studying hair cell-specific gene expression. However, these methods require that tissues are dissociated, the processing for which can lead to changes in gene expression prior to RNA extraction. To bypass this problem, we have developed a transgenic zebrafish model to evaluate the translatome of the inner ear and lateral line hair cells in their native tissue environment; the *Tg(myo6b:RiboTag)* zebrafish. This model expresses both GFP and a hemagglutinin (HA) tagged *rpl10a* gene under control of the *myo6b* promoter (*myo6b:GFP-2A-rpl10a-3xHA*), resulting in HA-tagged ribosomes expressed specifically in hair cells. Consequently, intact zebrafish larvae can be used to enrich for actively translated hair cell mRNA via an immunoprecipitation protocol using an antibody for the HA-tag (similar to the RiboTag mice). We demonstrate that this model can be used to reliably enrich for actively translated zebrafish hair cell mRNA. Additionally, we perform a global hair cell translatome analysis using RNA-Seq and show enrichment of known hair cell expressed transcripts and depletion of non-hair cell expressed transcripts in the immunoprecipitated material compared with mRNA extracted from whole fish (input). Our results show that our model can identify novel hair cell expressed genes in intact zebrafish, without inducing changes to gene expression that result from tissue dissociation and delays during cell sorting. Overall, we believe that this model will be highly useful for studying changes in zebrafish hair cell-specific gene expression in response to developmental progression, mutations, as well as hair cell damage by noise or ototoxic drug exposure.

## Introduction

Hearing loss is a genetically heterogeneous disorder, with mutations in over 150 genes estimated to underlie genetic non-syndromic hearing deficits (Van Camp and Smith, 2017). Of these, a large proportion affect genes that are preferentially expressed in the sensory cells of the inner ear, namely the mechanosensory hair cells (HCs) (Elkon et al., 2015). Zebrafish have served as an excellent model system for functional analysis of genes in HC function, as they possess HCs both in the inner ear as well as within an external lateral line system, are easy to manipulate genetically, and generate large numbers of progeny within a short gestational period (Nicolson, 2005, 2017; Erickson and Nicolson, 2015). However, the study of the molecular changes induced by manipulation of HC-expressed genes in zebrafish has been limited due to a paucity of models that allow cell type-specific molecular analysis of changes in gene expression. Specifically, across vertebrate species, the auditory, vestibular, and lateral line sensory organs are comprised of a variety of cell types, of which HCs make up only a small percentage (Hertzano and Elkon, 2012; Jiang et al., 2014; Matern et al., 2017). Therefore, due to their relative scarcity, cell type-specific approaches such as manual cell sorting, fluorescence activated cell sorting (FACS), or single cell RNA-Seq (scRNA-Seq) are necessary to analyze gene expression in HCs. These methods have been used in both mice and zebrafish to analyze HC gene expression changes that occur in mutant animals, during development and regeneration, or after exposure to noise or ototoxic drugs (McDermott et al., 2007; Hertzano and Elkon, 2012; Jiang et al., 2014; Steiner et al., 2014; Burns et al., 2015; Elkon et al., 2015; Scheffer et al., 2015). However, both scRNA-Seq and cell sorting-based techniques require dissociation of tissues to obtain a single cell suspension. Tissue dissociation can induce significant cellular stress due to loss of lateral inhibition and cell-cell contact, and combined with the prolonged time associated with tissue processing, may lead to confounding changes in gene expression (Sanz et al., 2009; Gay et al., 2013, 2014).

To avoid dissociation-induced molecular changes, recent studies have developed techniques in both mice and zebrafish models to extract RNA from specific cell types within intact organs (Heiman et al., 2009; Sanz et al., 2009; Gay et al., 2013; Tryon et al., 2013; Erickson and Nicolson, 2015; Roh et al., 2017). These approaches rely on pulldown of RNA from a cell type of interest via tagged ribosomes, or directly tagged RNA. As an example, the RiboTag mouse model expresses a component of the 60S subunit of the ribosome with a C-terminal hemagglutinin tag (RPL22-HA) (Sanz et al., 2009). Using this model, Cre-induced expression of RPL22-HA can be used to capture actively translated RNA from a cell type of interest via immunoprecipitation. The BACarray and NuTRAP mice models work in a similar way to RiboTag, however ribosomes are tagged with a green fluorescent protein (GFP). Additionally, the NuTRAP mice co-express nuclear tagging proteins that allow for concomitant epigenetic profiling of a Cre expressing cell type. In 2013, Tryon et al. also introduced several RiboTag/TRAP models to study cell type-specific gene expression in zebrafish (Tryon et al., 2013). Similar to the BACarray and NuTRAP mouse models, these zebrafish models rely on tagging the 60S ribosomal subunit through non-inducible tissue-specific expression of an *rpl10a*-GFP fusion gene. Collectively, in mouse and zebrafish, RiboTag, BACarray, and NuTRAP can be used for cell type-specific isolation of RNA via pulldown of labeled ribosomes. In these models, the immunoprecipitated RNA is enriched for actively translated transcripts, and is referred to as the “translatome” rather than the whole cellular transcriptome.

An alternative method to ribosomal pulldown is to isolate cell type-specific RNA from a complex tissue environment by thiouracil (TU) tagging. This method relies on tissue specific expression of uracil phosphoribosyltransferase (UPRT), an enzyme capable of integrating 4-thiouracil into newly synthesized RNA, which can then be affinity purified. Both mice and zebrafish TU-tagging models have been developed, and rather than obtaining only actively translated RNA as in the RiboTag/TRAP models, this method allows for capture of all newly synthesized RNA within a cell type of interest (i.e., the transcriptome) after the application of 4-thiouracil (Gay et al., 2013, 2014; Erickson and Nicolson, 2015). As with the ribosomal pulldown techniques, affinity purified RNA is enriched for the cell type of interest, and also contains some RNA from other tissues. In zebrafish, TU-tagging has been used to identify HC expressed transcripts. However, this model was only able to identify a small number of genes, and only those genes with very high expression levels in HCs were found to be significantly enriched in the immunoprecipitated RNA compared to input (Erickson and Nicolson, 2015).

To more effectively isolate RNA from zebrafish HCs, we have developed a transgenic zebrafish RiboTag model to evaluate the translatome of zebrafish inner ear and lateral line HCs; the *Tg(myo6b:GFP-2A-rpl10a-3xHA)* zebrafish [from here on referred to as *Tg(myo6b:RiboTag)*]. This model carries a construct to drive expression of both an HA-tagged Rpl10a protein and GFP in HCs under control of the HC-specific *myo6b* promoter (Seiler et al., 2004; Obholzer et al., 2008). To our knowledge, this is the first zebrafish model to allow for HC-specific gene expression analysis via two methods: (1) tissue dissociation and cell sorting based on GFP expression, and (2) immunoprecipitation of HA-tagged ribosomes to enrich for HC expressed transcripts. We use both RT-qPCR and RNA-Seq to analyze gene expression in our *Tg(myo6b:RiboTag)* model. We show that immunoprecipitated RNA from our *Tg(myo6b:RiboTag)* model is significantly enriched for known HC expressed transcripts, indicating that this model is effective in enriching for the HC translatome. Additionally, a comparison of our translatome dataset with a previously published zebrafish HC transcriptome dataset (generated using sorted HCs) shows that similar gene expression results can be obtained using the *Tg(myo6b:RiboTag)* model without cell sorting. Finally, we use the *Tg(myo6b:RiboTag)* model to identify novel HC expressed transcripts, and demonstrate that RiboTag immunoprecipitation helps to avoid gene expression changes that are induced by dissociation. Overall, we believe that this model will be highly useful for studying the normal development and function of zebrafish HCs, as well as changes to HC gene expression in response to different conditions.

## Materials and methods

### Zebrafish husbandry

Zebrafish were grown at 28°C in E3 embryo media (5 mM NaCl, 0.17 mM KCl, 0.33 mM CaCl_2_, and 0.33 mM MgSO_4_) using standard methods. Work performed at the National Institute of Health was approved by the NIH Animal Use Committee under animal study protocol #1362-13. At the University of Maryland School of Medicine (UMSOM), all procedures involving animals were carried out in accordance with the NIH Guide for the Care and Use of Laboratory Animals and have been approved by the Institutional Animal Care and Use Committee at the University of Maryland, Baltimore (protocol numbers 0514001 and 1116003).

### Vector construction and generation of the *Tg(myo6b:GFP-2A-rpl10a-3xHA)* zebrafish

Plasmid construction was based on the Tol2/Gateway zebrafish kit (Kwan et al., 2007). The *pME-GFP-2A-rpl10a-3xHA* was a generous gift from Dr. Brant Weinstein at the NIH. The p5E-*pmyo6b* entry clone used to drive expression in hair cells has been described previously (Kindt et al., 2012). These two clones were used along with the tol2 kit gateway clones p3E-polyA (#302) and pDest (#394) to create the *myo6b:GFP-2A-rpl10a-3xHA* expression construct. To generate a stable transgenic fish line, plasmid DNA at 50 ng/μL and tol2 transposase mRNA at 20 ng/μL were injected into zebrafish embryos as previously described to create *Tg(myo6b:GFP-2A-rpl10a-3xHA)*^*idc*10^(Kwan et al., 2007). Each line was grown to the F1 generation and outcrossed to confirm single copy integration.

### Immunohistochemistry and confocal imaging

Immunohistochemistry was performed on whole-mount larvae. Wildtype or transgenic larvae were fixed with 4% paraformaldehyde in phosphate buffered saline (PBS) for 4 h at 4°C. After 5 × 5 min washes in PBS, followed by a 5 min wash in H_2_O, larvae were permeabilized with ice cold acetone (at −20°C) for 5 min. Larvae were then washed in H_2_O for 5 min, followed by a 5 × 5 min washes in PBS, and then blocked overnight with PBS containing 2% goat serum and 1% bovine serum albumin (BSA). A primary rat anti-HA antibody (Roche) was diluted at 1:750 in PBS containing 1% BSA, and larvae were incubated in the solution for 4 h at room temperature. After 5 × 5 min washes in PBS to remove the primary antibody, an Alexa 568 secondary antibody diluted at 1:1,000 (Life Technologies) was added in PBS containing 1% BSA and incubated overnight at 4°C. After 5 × 5 min washes in PBS to remove the secondary antibody, larvae were rinsed in H_2_O and mounted in Prolong Gold (Life Technologies). Fixed samples were imaged on an inverted Zeiss LSM 780 laser-scanning confocal microscope with a 63 × 1.4 NA oil objective lens. Excitation wavelengths of 488 and 546 nm were used to excite GFP and Alexa 568, respectively.

### *Tg(myo6b:RiboTag)* translatome immunoprecipitation

The *Tg(myo6b:RiboTag)* immunoprecipitation protocol was modified from the RiboTag immunoprecipitation protocol described in Sanz et al. (2009). Briefly, GFP^+^ *Tg(myo6b:RiboTag)* zebrafish larvae were euthanized at 5 days post fertilization (dpf) using MS-222/Tricaine and rinsed with system water. Groups of fifty larvae were then either flash frozen and stored at −80°C or used immediately for immunoprecipitation. Larvae were resuspended and homogenized in 1 mL of supplemented homogenization buffer (50 mM Tris-HCl pH.7, 100 mM KCl, 12 mM MgCl_2_, 1% Nonidet P-40, 1 mM 1,4-Dithiothreitol, 1X protease inhibitor cocktail, 200 U/mL RNAsin, 100 μg/mL cycloheximide, 1 mg/mL heparin) by douncing on ice. Homogenates were spun at 10,000 g for 10 min at 4°C to remove particulates, and a small sample of clear supernatant was reserved for total RNA isolation (input control, IN). Remaining supernatant was incubated with 5 μg HA antibody (BioLegend) at 4°C under gentle rotation for 4–6 h. After incubation, supernatants were incubated with 300 μL of rinsed Invitrogen Dynabeads Protein G magnetic beads (Thermo Fisher) overnight, rotating. Following incubation, bound beads were rinsed three times with 800 μL high salt buffer (50 mM Tris-HCl pH.7, 300 mM KCl, 12 mM MgCl_2_, 1% Nonidet P-40, 1 mM 1,4-Dithiothreitol, 100 μg/mL cycloheximide) at 4°C for 10 min, rotating. After washing, 350 μL of buffer RLT from the RNeasy Plus Micro kit (Qiagen) was added to the bound beads or reserved input sample and vortexed for 30 s to dissociate bound RNA. RNA was then extracted according to manufacturer's instructions (using 16 μL of nuclease free water for elution) and stored at −80°C. RNA quality and concentration was assessed using the Agilent RNA Pico kit (Agilent Technologies) at the UMSOM Genomics Core Facility.

### Fluorescence activated cell sorting

Zebrafish dissociation and FACS were performed as previously described (Elkon et al., 2015). Briefly, approximately 150 *Tg(myo6b:RiboTag)* larvae per replicate were euthanized at 5 dpf and dissociated in cold trypsin-EDTA solution (0.5 g/L trypsin, 0.2 g/L EDTA, Sigma) by trituration with a p1000 pipette tip on ice for 20 min. Dissociation was then halted by adding HBSS supplemented with 10% fetal bovine serum (FBS) and 100 μg/mL DNaseI. Cells were filtered through a 70 μm cell strainer (Fischer Scientific) and pelleted by centrifugation at 2,000 rpm for 10 min at 4°C. Cells were washed once in HBSS, resuspended in HBSS supplemented with 10% FBS, and filtered into glass tubes through a 35 μm cell strainer (Falcon). Flow cytometry analyses were performed at the University of Maryland Marlene and Stewart Greenebaum Comprehensive Cancer Center Flow Cytometry Shared Service. Samples of GFP positive and negative cells (HCs and the rest of the fish) were collected using a BD FACSAria II (BD Biosciences), and a small aliquot of each sorted population was re-analyzed to determine cell purity. RNA was extracted from sorted cells using Trizol LS Reagent (Thermo Fischer Scientific) and the Direct-zol™ RNA MiniPrep Plus (Zymo Research).

### RT-qPCR

RT-qPCR was performed as described previously with minor modifications (Matern et al., 2017). RNA from 5 dpf *Tg(myo6b:RiboTag)* zebrafish input and IP samples, or sorted cells, was reverse-transcribed using the Maxima First Strand cDNA Synthesis Kit (Thermo Fisher Scientific), and qPCR was performed using the Maxima SYBR Green/ROX qPCR Master Mix (Thermo Fisher Scientific). To account for low levels of RNA obtained from sorted cells, a preamplification step using PerfeCTa PreAmp Supermix (Quantbio) was added for the RNA-Seq validation and immediate early gene expression experiments. Expression values were normalized to *actb1* expression (see Supplementary Table 4 for primer sequences).

### RNA sequencing and informatics

RNA from 5 dpf *Tg(myo6b:RiboTag)* zebrafish IN and IP samples was submitted in biological quadruplicates for RNA-Seq at the UMSOM Institute for Genome Sciences. Only samples with RNA integrity numbers (RIN) > 8 were used for sequencing. Libraries were prepared from 25 ng of RNA using the TruSeq RNA Sample Prep kit (Illumina) per manufacturer's instructions, with the exception of an additional PCR cycle. Samples were sequenced on an Illumina HiSeq 4000 with a 75 bp paired-end read configuration. Between 100 and 150 million reads were obtained for each sample, and reads were aligned to the zebrafish genome (*Danio rerio*.GRCz10) using TopHat version 2.0.8 (maximum number of mismatches = 2; segment length = 30; maximum multi-hits per read = 25; maximum intron length = 50,000) (Kim et al., 2013). The number of reads that aligned to the predicted coding regions were determined using HTSeq (Anders et al., 2015), and only genes with CPM (reads count per transcripts per million mapped reads) values > 0.01 in all IN and IP replicates were called as expressed (17,164 genes). One IP sample had a high intergenic content suggestive of DNA contamination and was excluded from the analysis. See Supplementary Table 1 for alignment statistics. Significant differential expression was assessed using DEseq (Anders and Huber, 2010). RNA-Seq data were submitted to the Gene Expression Omnibus database (GEO accession GSE102861), as well as the gEAR Portal (UMgEAR.org). Gene ontology of highly enriched and depleted gene sets was performed using the Gene Ontology (GO) database (http://www.geneontology.org) (Harris et al., 2004). For HC enriched and depleted gene sets, top branches of GO terms are shown. Anatomical structure enrichment was performed using the Zebrafish Expression Ontology of Gene Sets (ZEOGS) tool (Prykhozhij et al., 2013), with a corrected *p*-value cutoff of 0.10. Sorted HC dataset transcript IDs from Steiner et al. were converted to gene IDs using bioDBnet (https://biodbnet-abcc.ncifcrf.gov) (Mudunuri et al., 2009).

## Results

### Generation of *Tg(myo6b:GFP-2A-rpl10a-3xHA)* zebrafish to create the *Tg(myo6b:RiboTag)* model

**Figure 1:**
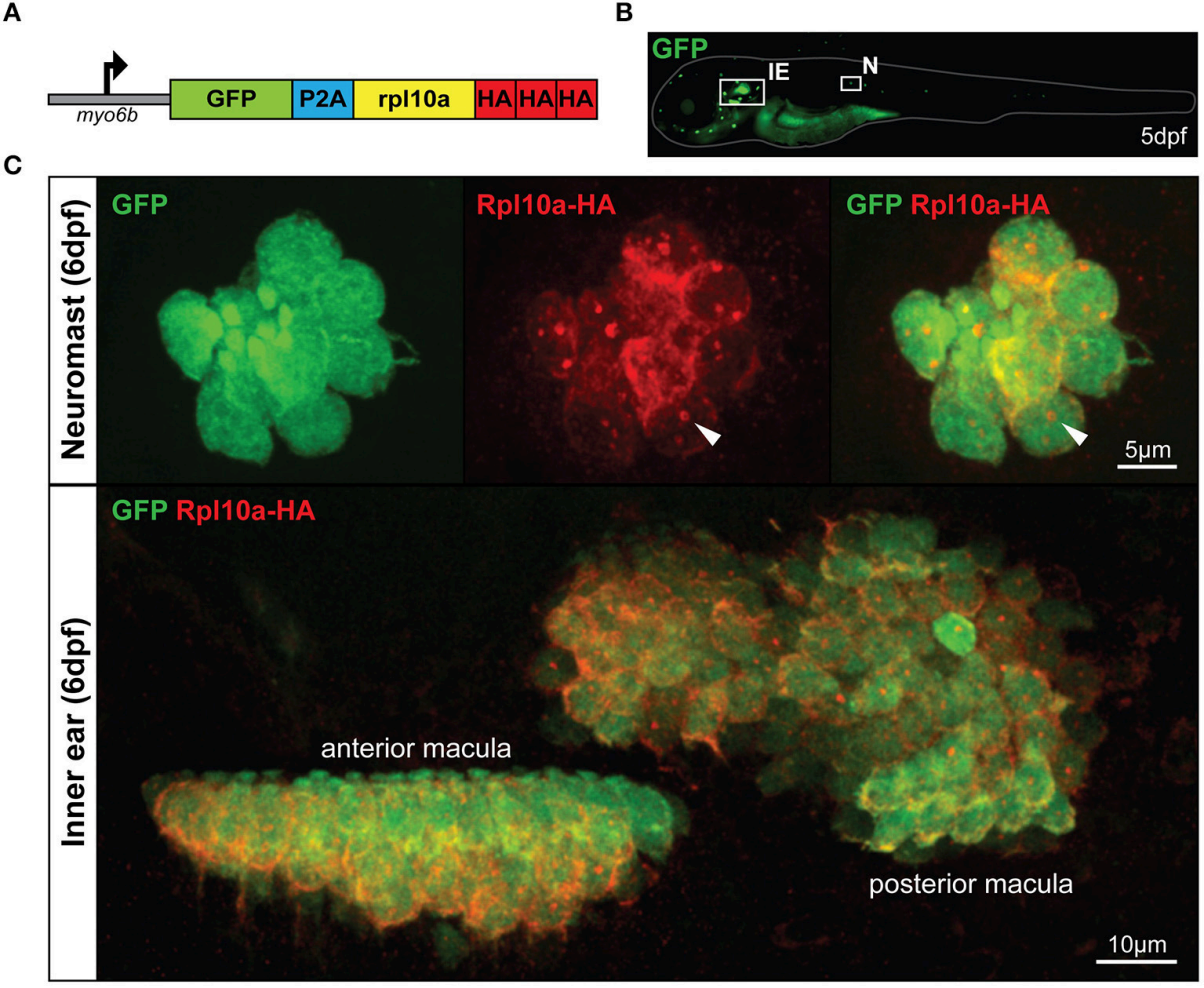
The *Tg(myo6b:RiboTag)* zebrafish. **(A)** Schematic representation of the GFP-2A-rpl10a-3xHA construct driven by the HC-specific *myo6b* promoter. **(B)** Representative image of a live *Tg(myo6b:RiboTag)* zebrafish at 5 dpf showing GFP expression in the inner ear (IE) and lateral line neuromasts (N). **(C)** Immunohistochemistry using an HA antibody showing that Rpl10a-HA expression overlaps with GFP expression specifically within the HCs of the inner ear and neuromasts. White arrows denote nucleolar Rpl10a-HA staining.

To create a RiboTag zebrafish model that would allow for enrichment of the inner ear and lateral line HC translatome, we utilized the *rpl10a* gene, which has previously been used to effectively immunoprecipitate ribosomes in zebrafish via a GFP tag (Tryon et al., 2013). However, instead of an Rpl10a-GFP fusion protein, here we use an Rpl10a-3xHA fusion protein for the ribosomal pulldown. To drive Rpl10a-3xHA expression in HCs we utilized the HC-promoter *myo6b*, which has been shown to specifically drive expression of downstream genes in zebrafish inner ear and lateral line HCs (Obholzer et al., 2008). The construct is engineered to express Rpl10a-3xHA together with GFP bi-cistronically using the viral P2A peptide, resulting in the *myo6b:GFP-2A-rpl10a-3xHA* construct (Figure 1A). This construct was injected into zebrafish embryos at the one-cell stage to create a stable *Tg(myo6b:GFP-2A-rpl10a-3xHA)* transgenic line, referred to here as our *Tg(myo6b:RiboTag)* model. To confirm Rpl10a-3xHA was localizing properly in HCs, we immunostained our *Tg(myo6b:RiboTag)* zebrafish with an anti-HA antibody at 5 or 6 dpf, when HCs in the inner ear and along the lateral line have developed. As expected, we observed both GFP and HA signal localizing to the cytosol of inner ear and lateral line HCs (Figures 1B,C). Consistent with previously observed Rpl10a-GFP localization in *xef1*α>TRAP zebrafish (Tryon et al., 2013), the HA staining in the HCs of the *Tg(myo6b:RiboTag)* zebrafish also shows nucleolar localization (Figure 1C, white arrows), indicating that our model is localizing Rpl10a-3xHA properly.

### Ribosome immunoprecipitation isolates the HC translatome from *Tg(myo6b:RiboTag)* zebrafish

**Figure 2:**
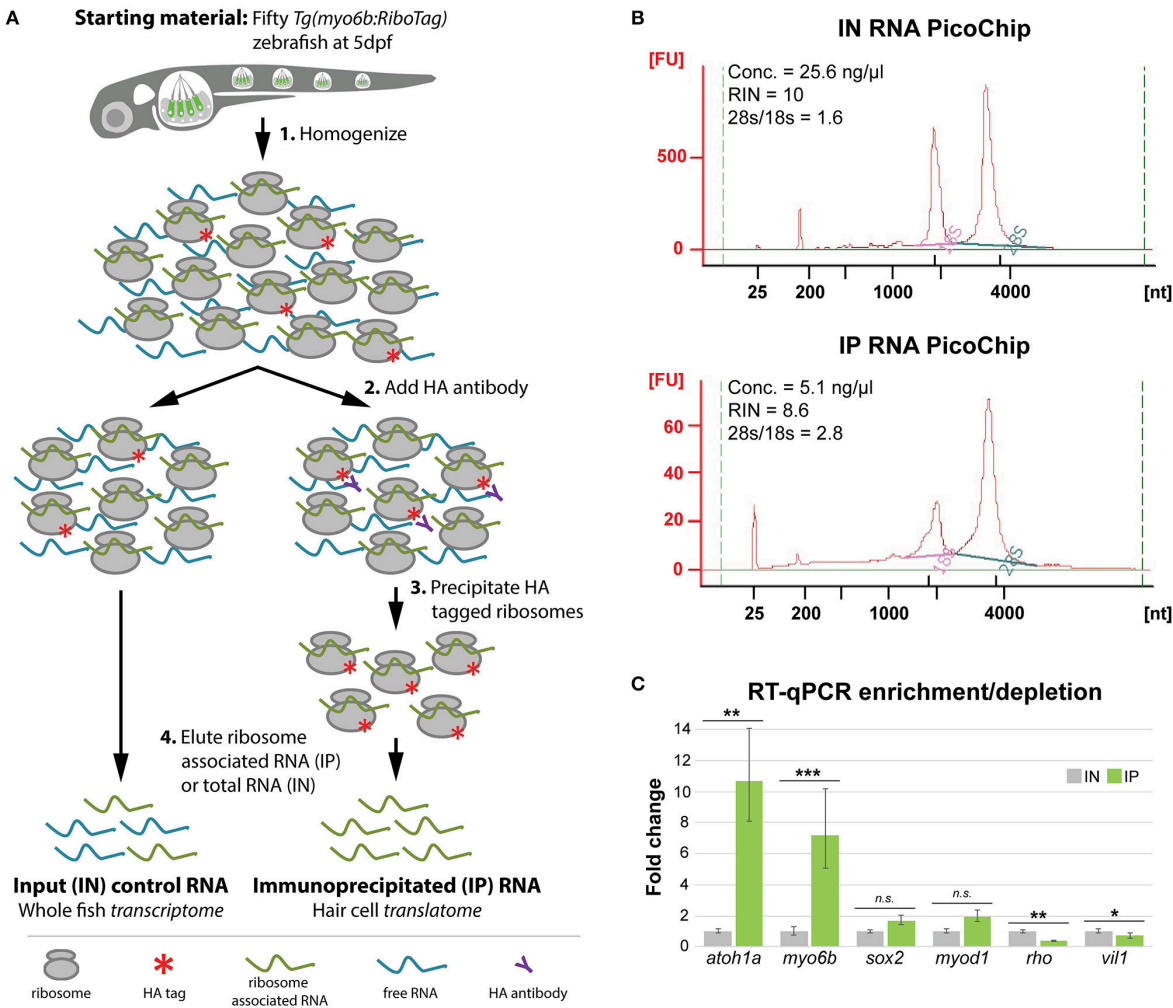
HC ribosome immunoprecipitation pulls down HC-specific transcripts. **(A)** Explanation of the HA-tagged ribosome immunoprecipitation protocol (see Methods for more information). **(B)** Agilent Bioanalyzer PicoChip outputs showing that input (IN) and immunoprecipitated (IP) RNA quality is comparable between sample types despite reduced levels of 18S rRNA in the IP samples. This observation is consistent with previous zebrafish ribosome immunoprecipitation protocols (Tryon et al., 2013). **(C)** RT-qPCR results showing that transcripts for known HC-expressed genes such as *atoh1a* and *myo6b* are significantly enriched by HA-tagged ribosome immunoprecipitation, whereas transcripts for genes not specifically expressed in HCs are either not significantly enrichened (*sox2, myod1*) or depleted (*rho, vil1*). Error bars represent fold change ± standard deviation, and statistical significance was assessed by two-tailed Welch's *t*-test (*n* = 10). *p-value < 0.05, **p-value < 0.01, ***p-value < 0.001.

In order to test whether the *Tg(myo6b:RiboTag)* zebrafish could be used to analyze gene expression of the inner ear and lateral line HCs, we next adapted the RiboTag immunoprecipitation protocol described in Sanz et al. (2009) to capture the HC translatome at 5 dpf (Figure 2A) using fresh or frozen embryos. We chose 5 dpf as the time point for our experiment, as this time point allows us to compare our results to other previously published HC gene expression datasets also generated using 5 dpf larvae. This technique yields ~50 ng of immunoprecipitated RNA (IP, average RNA concentration = 3.5 ± 2.3 ng/μL in 16 μL elution volume) and 660 ng of input control RNA (IN, average RNA concentration = 41.3 ± 24.6 ng/μL in 16 μL elution volume) per fifty homogenized *Tg(myo6b:RiboTag)* larvae (*n* = 19 IPs). Because of the amount of homogenate used for immunoprecipitation, these quantities of RNA correspond to ~330 ng of input RNA and ~1 ng immunoprecipitated RNA per larvae. Interestingly, while analysis of the IN samples showed characteristic ratio of 1.6–2 between the 28s to 18s rRNA, representing intact RNA components of the 60S and 40S ribosomal subunits respectively, the IP samples showed ratios >2.5, indicating reduced levels of 18s rRNA (Figure 2B). This observation is consistent with the zebrafish ribosomal immunoprecipitation results in Tryon et al. (2013), and is thought to be a result of tagging the 60S subunit of the ribosome (of which Rpl10a is a component). Overall, these results indicate that high quality RNA in amounts suitable for downstream analyses such as RT-qPCR and RNA sequencing (RNA-Seq) can be obtained using the *Tg(myo6b:RiboTag)* fish along with our immunoprecipitation protocol.

After establishing our protocol, we next aimed to validate whether the immunoprecipitation step was efficiently pulling down RNA from HCs. For this analysis, we used RT-qPCR to determine the relative transcript abundance of known HC expressed genes between IN and IP samples. As expected, immunoprecipitation of HA-tagged ribosomes resulted in significant enrichment of transcripts for the HC expressed genes *atoh1a*, which encodes a transcription factor necessary for HC development, and *myo6b*, the promoter of which is used to drive *rpl10a-3xHA* expression in this model (Figure 2C) (Seiler et al., 2004; Millimaki et al., 2007). We did not observe significant differences in transcript abundance for the supporting cell expressed gene *sox2* or the muscle expressed gene *myod1*. However, we observed significant depletion of the eye specific gene *rho* and gut specific gene and *vil1* in the RNA obtained from the IP compared to the IN. These results indicate that the protocol adapted for the *Tg(myo6b:RiboTag)* ribosome immunoprecipitation is able to specifically enrich for transcripts of HC expressed genes, while also depleting, to a varying extent, transcripts expressed in other cell types.

### RNA sequencing of IP and in samples from *Tg(myo6b:RiboTag)* zebrafish

**Figure 3:**
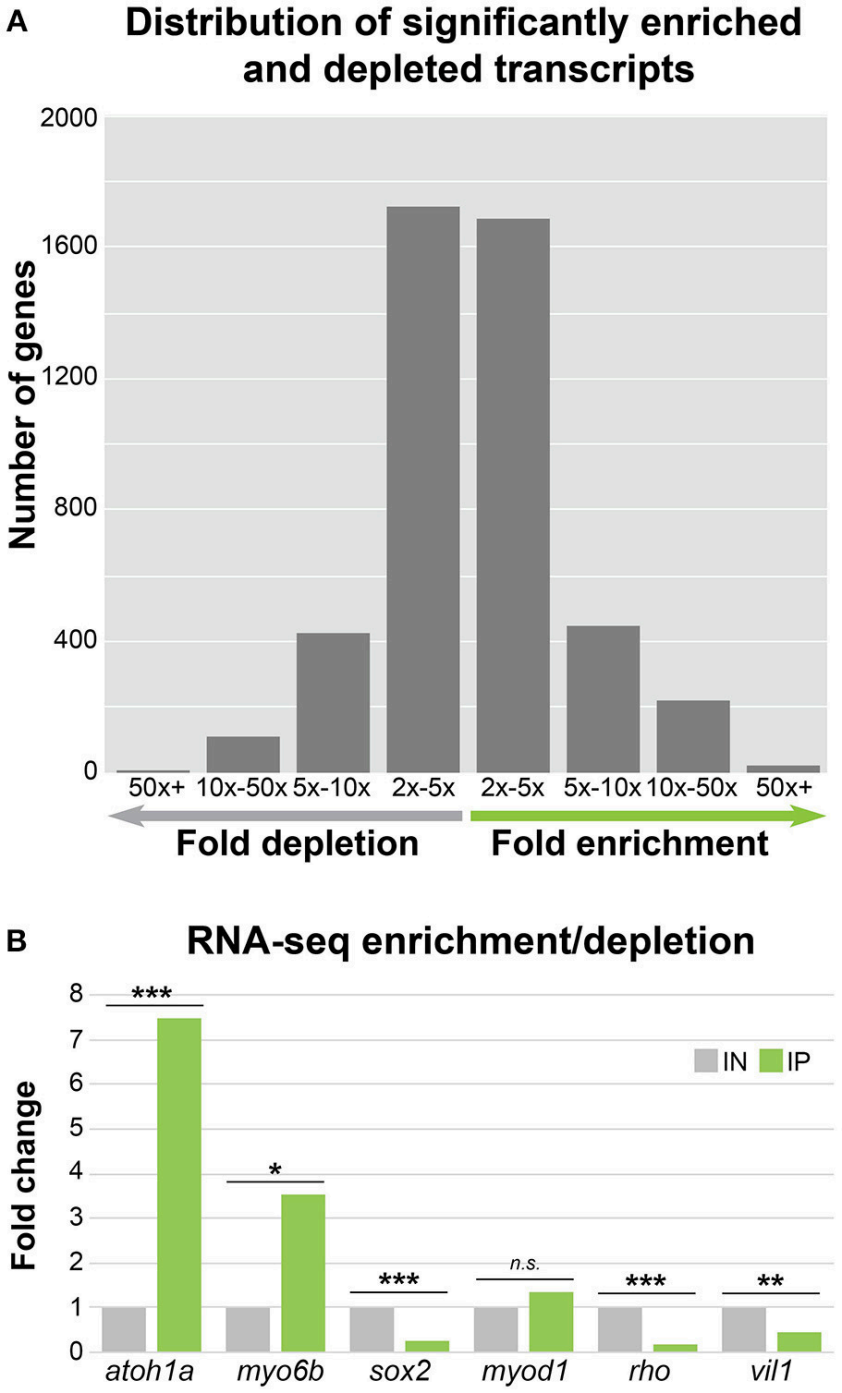
HC ribosome immunoprecipitation can reliably detect HC enriched and depleted transcripts by RNA-Seq. **(A)** Bar graph showing the distribution of significantly enriched and depleted transcripts in the IN vs. IP samples binned by fold change range. For genes with depleted transcripts in the IP samples, the number of genes per bin is as follows: 2x − 5x = 1726, 5x − 10x = 422, 5x − 10x = 109, and 50x + = 1. For IP enriched gene transcripts, the number of genes per bin is as follows: 2x − 5x = 1685, 5x − 10x = 450, 5x − 10x = 223, and 50x + = 21. **(B)** RNA-Seq fold change enrichment and depletion of HC expressed and non-expressed genes replicates the results obtained by RT-qPCR (Figure 2C). Statistical significance was assessed using DEseq (see Methods). *FDR < 0.05, **FDR < 0.01, ***FDR < 0.001.

After confirming the efficiency of immunoprecipitation protocol, we next sought to perform an unbiased and systematic analysis of the utility of the *Tg(myo6b:RiboTag)* model as a tool to study the zebrafish HC translatome. We therefore performed RNA-Seq on IP and IN samples in atleast three biological replicates from 5 dpf *Tg(myo6b:RiboTag)* zebrafish. In total, 17,164 genes were detected as expressed in our IN and IP samples based on our criteria (IN and IP counts per million [CPM] > 0.01 in all samples), allowing for a direct comparison of expression levels (see Supplementary Data Sheet 1). Of these, transcripts for 2,379 genes were significantly enriched in IP samples compared to IN (fold change > 2, IP CPM > 1, false discovery rate [FDR] < 0.05), and transcripts of 2,258 genes were significantly depleted in IP samples compared to IN (fold change < 0.5, IN CPM > 1, FDR < 0.05) (Figure 3A). As an internal control, we first analyzed transcript enrichment and depletion of the same HC and non-HC expressed genes used in our RT-qPCR analysis (Figure 2C) and saw parallel results (Figure 3B). Our RNA-Seq analysis also found that transcripts for the known HC genes *atoh1a* and *myo6b* are significantly enriched in the IP samples compared to IN.

As shown in Figure 3A, the majority of transcripts found to be significantly enriched or depleted in the IP compared to the IN samples had a two to five-fold-change in transcript abundance. This large number of genes with small differences in transcript abundance between IP and IN could be attributed to the likelihood that many HC-expressed genes are also expressed in other cell types. Because our protocol isolates RNA from whole, intact larvae, concomitant enrichment and depletion of RNA expressed in both HCs and other cell types could lower the overall fold change values for HC enrichment and depletion. Therefore, in order to restrict our analyses to genes with a preferential expression in HCs compared to other tissues, we focused on genes with high fold-change transcript enrichment in the IP samples. A gene ontology (GO) analysis of the genes with five-fold or greater transcript enrichment in IP compared to IN samples (*n* = 694) identified “detection of mechanical stimulus” as a top enriched GO term (Table 1, GO associated with HC function in bold). This same analysis was also performed on the genes with five-fold or greater depletion in the IP compared to IN samples (*n* = 532), resulting in top GO terms including “detection of light stimulus” and “visual perception” (Table 2, in bold). Together with our RT-qPCR, our RNA-Seq, and GO analysis of five-fold enriched transcripts support that our *Tg(myo6b:RiboTag)* model and immunoprecipitation protocol can enrich for the zebrafish HC translatome.

**Table 1:** Gene ontology analysis of hair cell enriched genes.

**GO biological process complete**	**# in Reference**	**# in input**	**# expected**	**Fold enrichment**	**FDR**	**Genes**
miRNA mediated inhibition of translation	9	5	0.21	24.32	0.00252	*trim71 tnrc6b tnrc6a tnrc6c1 ago2*
Skeletal muscle contraction	23	5	0.53	9.52	0.0373	*CU929259 tnni1c tnnc1b CU929259 tnni1d*
**detection of mechanical stimulus**	25	5	0.57	8.76	0.0496	*tmc2a loxhd1a loxhd1b dennd5a lhfpl5a*
**sensory perception of mechanical stimulus**	48	7	1.1	6.37	0.0283	*tmc2a pcdh15b atp2b1a lhfpl5a mecp2 BX572619 dcdc2b*
**neuromast development**	62	8	1.42	5.64	0.0237	*atoh1a pcsk5a slc12a5b atp2b1a pho BX572619 erbb2 dcdc2b*
Steroid hormone mediated signaling pathway	77	9	1.76	5.11	0.0198	*vdra abhd2a pparda thrab rorb rorcb nr6a1b thrb nr0b2a*
mRNA metabolic process	299	19	6.84	2.78	0.0182	*celf4 ptbp3 qkib rbbp6 rbmx2 crnkl1 snrnp70 tnrc6a nova2 rbm25a exosc7 qkia kiaa0907 exosc3 coil snrpa aqr dicer1 ago2*
Transcription, DNA-templated	841	43	19.24	2.23	0.00093	*hdac4 hoxb1a pou6f2 vdra nfia rest stat2 hoxb3a foxh1 brf2 pparda hoxc6b bhlhe41 hoxa1a thrab rorb ncoa1 gtf2a1l nfat5a eed rorcb mef2b nrarpa mef2aa nr6a1b clocka med19a pou2f1b tcf3a jarid2b thrb onecut2 BX005395 twistnb onecut1 mecp2 ccnd1 hoxa1a znf367 nr0b2a pou2f2a asxl2 nfic*
Regulation of transcription, DNA-templated	1703	71	38.97	1.82	0.00092	*hdac4 hoxb1a nkap pou6f2 pde8b vdra nfia rest mkl2a stat2 hoxb3a foxh1 brf2 pparda hoxc6b has2 tcf3a bhlhe41 hoxa1a thrab gfi1aa trps1 rorb atoh1a foxj3 ncoa1 crebrf zfhx4 fosl1a nfat5a eed rfx7 rorcb tomm20a ches1 mef2b nrarpa CU633479 mef2aa nr6a1b clocka hmbox1b med19a pou2f1b tcf3a jarid2b thrb BX511021 cica onecut2 pbxip1b zfhx3 BX005395 onecut1 mecp2 rbpja foxb1b ccnd1 hnf1a hoxa1a tbl1xr1b mycbp znf367 nr0b2a pou2f2a crtc1a ago2 asxl2 nfic dot1l rbpjb*

**Table 2:** Gene ontology analysis of hair cell depleted genes.

**GO biological process complete**	**# in Reference**	**# in Input**	**# expected**	**Fold enrichment**	**FDR**	**Genes**
Antibiotic catabolic process	4	3	0.07	41.55	0.024	*esd cat amdhd2*
ATP synthesis coupled electron transport	43	7	0.78	9.02	0.00537	*AC024175.9 (*associated with *mt-nd4l mt-cyb mt-nd2 mt-co1 mt-nd4 mt-nd5)*
**Detection of light stimulus**	38	6	0.69	8.75	0.0176	*opn1mw1 gnat2 rho lamc1 opn1sw1 (*also associated with *opn1sw2)*
Pyruvate metabolic process	43	6	0.78	7.73	0.0275	*hkdc1 aldoca eno2 pdha1b aldoaa pkmb*
ATP biosynthetic process	64	8	1.16	6.93	0.00767	*hkdc1 aldoca eno2 AC024175.9 (*associated with *mt-atp6) aldoaa BX901937 pkmb*
**Visual perception**	97	12	1.75	6.85	0.00022	*opn1mw1 kera gnat2 rho lamc1 cryaa crx aoc2 irbp vsx2 opn1sw1 (*also associated with *opn1sw2)*
Proton transmembrane transport	57	7	1.03	6.8	0.0194	*atpv0e2 AC024175.9 (*associated with *mt-co1 mt-atp6) BX901937 atp6v0b atp6v1b2*
Regulation of cell growth	97	9	1.75	5.14	0.0179	*igfbp7 osgn1 ncaldb chrna1 casp9 epb41l3b lamtor2 dpysl2b arl3l1*
Coenzyme biosynthetic process	116	9	2.1	4.3	0.0436	*aldoca eno2 spra mat2al pdha1b aldoaa ndufa9b pkmb*
Nucleobase-containing compound catabolic process	153	11	2.77	3.98	0.0239	*hkdc1 aldoca eno2 upf3a smg5 pcid2 polr2gl aldoaa dis3 dnase1l3l pkmb*
Cellular protein localization	497	23	8.99	2.56	0.0115	*vps11 copb1 ap2m1b ap4e1 smg5 tomm40l vps29 pcid2 glrbb chrna1 atg9a copa wipi2 epb41l3b BX901937 grpel2 nmd3 hsc70 sx1b napgb pttg1ipb sec24d lamtor2*
Protein transport	530	24	9.59	2.51	0.0113	*arcn1b vps11 rab4a copb1 ap2m1b ap4e1 smg5 tomm40l vps29 pcid2 rab10 tsg101a atg9a copa tvp23b BX901937 (*associated with *Zgc:165520 vps37c) grpel2 nmd3 snx1b napgb jagn1a pttg1ipb sec24d*
System development	2,933	79	52.94	1.49	0.0437	*tfap2b cdh7 tyrp1b inpp5b wif1 vps11 rab4a ecrg4b igfbp7 copb1 slc2a2 slc35b2 col17a1b ponzr1 epb41b ncaldb fosab rab10 slc4a1a PPP1CC pou4f2 slc33a1 dacha ruvbl2 chrna1 snapc2 gdpd3a celsr2 copa rapsn ephb4a smyd1b casp9 lingo2a inpp5kb mcm3 olfm2a aldoaa grhl2a rtn4r BX470189 fez1 lamc1 dhps cryaa itm2bb id4 klhl40a camk1db crx lrfn4b rel lhx1a jagn1a inab scinla lamb2 padi2 anos1a slitrk6 zic6 CRIP2 plppr1 pttg1ipb sec24d polr3b ugdh SLITRK1 atp6v0b dpysl2b mab21l2 pou3f1 arl3l1 hpse lrrn1 PDCL3 dnase1l3l spry4 six3a*

### Immunoprecipitation enriches for known HC expressed transcripts

We next wanted to assess the efficiency of our model for enriching for other genes known to be expressed in zebrafish HCs. Therefore, we analyzed our transcripts with five-fold or greater enrichment (*n* = 694) and depletion (*n* = 532), from here on name “enriched” and “depleted” gene sets, using the Zebrafish Expression Ontology of Gene Sets (ZEOGS) tool (Prykhozhij et al., 2013). This online tool can be used to detect overrepresented anatomical structures based on known gene expression patterns from the Zebrafish Information Network (ZFIN) (Howe et al., 2013). When analyzing the enriched gene set, the top result for overrepresented anatomy is “neuromast,” and also includes the inner ear HC types “hair cell anterior macula” and “hair cell posterior macula” (Table 3). We also performed a ZEOGS analysis on the highly depleted genes, resulting in the top term of “retinal photoreceptor layer” and no results related to the inner ear or neuromast hair cells (Supplementary Table 2). This indicates that the *Tg(myo6b:RiboTag)* immunoprecipitation is capturing transcripts of genes experimentally validated to be expressed in neuromast and inner ear HCs.

**Table 3:** ZEOGS analysis of HC enriched transcripts.

**Anatomical term**	**Corrected *p*-value**	**Genes**
Neuromast	0.00052	*cabp2b morn3 pcsk5a rorb s100t atp2b1a gfi1aa otofa wasa pho bdnf pvalb8 tmc2a tmc2b s100s myclb atoh1a*
Levator operculi	0.06849	*tnnc1b smyhc2*
Hair cell anterior macula	0.07032	*atp2b1a tmc2a otofa*
Hyohyoideus	0.07515	*myha tnnc1b smyhc2*
Olfactory epithelium	0.0832	*tnks1bp1 cnga3a s100t dlg2 bdnf s100s s100a1 elavl3*
Olfactory bulb	0.0915	*klf7a mef2aa fabp10b plxnb2b s100t dlg2 bdnf pvalb8 s100s cadm1b igdcc3*
Hair cell posterior macula	0.09839	*atp2b1a tmc2a*

Zebrafish have proved to be a highly useful animal model for studying the normal development and function of HCs, as well as for dissecting the mechanistic consequences of gene mutations that cause human hearing loss (Lieschke and Currie, 2007). For this reason, we next probed our RNA-Seq dataset to detect zebrafish homologs of known human syndromic and non-syndromic deafness causing genes reported on the Hereditary Hearing Loss Homepage (http://hereditaryhearingloss.org) (Van Camp and Smith, 2017). In total, we found that 36 deafness genes were significantly enriched in our zebrafish HC IP samples compared to IN (fold change > 2, *p*-value < 0.05), of which 27 remained significant after taking into account multiple testing (Table 4, bold values denote FDR < 0.05). Importantly, only 13 of these genes have been previously reported in the literature to be expressed in the developing zebrafish inner ear or HCs, which indicates that the *Tg(myo6b:RiboTag)* model is effective in identifying genes not previously known to be expressed in zebrafish HCs.

**Table 4:** Fold change enrichment of human deafness gene homologs in *Tg(myo6b:RiboTag)* IP samples.

**Ensembl ID**	**Zebrafish gene name**	**Human homolog**	**Human disease**	**Fold enrichment in zebrafish HCs**	***p*-value**	**FDR**	**Previously reported expression in zebrafish HCs**
ENSDARG00000074638	*loxhd1b*	*LOXHD1*	AR non-syndromic deafness	77.35484	3.01E−57	**1.6E**–**53**	NA
ENSDARG00000094738	*loxhd1a*	*LOXHD1*	AR non-syndromic deafness	47.78006	8.84E−20	**1.63E**–**17**	NA
ENSDARG00000056386	*tmc1*	*TMC1*	AD and AR non-syndromic deafness	15.46066	0.00102	**0.007132**	Maeda et al., 2014; Erickson et al., 2017
ENSDARG00000053074	*gipc3*	*GIPC3*	AR non-syndromic deafness	15.4136	4.61E−26	**1.7E**–**23**	NA
ENSDARG00000105391	*si:cabz01059983.1*	*STRC*	AR non-syndromic deafness	14.00558	7.37E−16	**7.78E**–**14**	NA
ENSDARG00000056458	*lhfpl5b*	*LHFPL5*	AR non-syndromic deafness	12.75537	2.74E−07	**5.53E**–**06**	NA
ENSDARG00000020581	*otofb*	*OTOF*	AR non-syndromic deafness	12.37504	4.49E−29	**2.6E**–**26**	Chatterjee et al., 2015
ENSDARG00000105434	*grxcr1*	*GRXCR1*	AR non-syndromic deafness	10.61186	4.21E−07	**8.07E**–**06**	NA
ENSDARG00000053692	*grxcr2*	*GRXCR2*	AR non-syndromic deafness	10.00353	0.011347	0.052075	NA
ENSDARG00000074742	*elmod3*	*ELMOD3*	AR non-syndromic deafness	7.635278	0.043045	0.145634	NA
ENSDARG00000053315	*tmprss3a*	*TMPRSS3*	AR non-syndromic deafness	7.302665	2.63E−15	**2.52E**–**13**	NA
ENSDARG00000008849	*ptprq*	*PTPRQ*	AR non-syndromic deafness	6.882463	0.030491	0.11237	NA
ENSDARG00000008127	*pcdh15b*	*PCDH15*	Usher syndrome type 1F and 1D, AR non-syndromic deafness	6.751992	6.03E−09	**1.81E**–**07**	Seiler et al., 2005; Maeda et al., 2014
ENSDARG00000076414	*espn*	*ESPN*	AR non-syndromic deafness	6.702256	0.00318	**0.018583**	NA
ENSDARG00000030832	*otofa*	*OTOF*	AR non-syndromic deafness	6.1553	6.87E−12	**3.71E**–**10**	Thisse and Thisse, 2004; Chatterjee et al., 2015
ENSDARG00000040046	*snai2*	*SNAI2*	Waardenburg syndrome	5.542076	1.21E−13	**9.15E**–**12**	NA
ENSDARG00000052277	*cabp2b*	*CABP2*	AR non-syndromic deafness	5.462416	1.59E−13	**1.18E**–**11**	Di Donato et al., 2013
ENSDARG00000045023	*lhfpl5a*	*LHFPL5*	AR non-syndromic deafness	5.390774	0.002062	**0.012931**	Erickson and Nicolson, 2015
ENSDARG00000075870	*triobpa*	*TRIOBP*	AR non-syndromic deafness	5.012163	6.12E−13	**4.04E**–**11**	NA
ENSDARG00000005335	*slc22a4*	*SLC22A4*	AR non-syndromic deafness	4.781316	0.000239	**0.002071**	NA
ENSDARG00000029482	*ush2a*	*USH2A*	Usher syndrome type 2A	4.61055	0.047798	0.157459	Blanco-Sánchez et al., 2014
ENSDARG00000105136	*ccdc50*	*CCDC50*	AD non-syndromic deafness	4.590251	9.01E−11	**3.98E**–**09**	NA
ENSDARG00000006385	*triobpb*	*TRIOBP*	AR non-syndromic deafness	3.836217	5.98E−08	**1.44E**–**06**	NA
ENSDARG00000042141	*myo6b*	*MYO6*	AD and AR non-syndromic deafness	3.512935	0.007429	**0.037159**	Seiler et al., 2005; McDermott et al., 2007
ENSDARG00000010192	*pax3a*	*PAX3*	Waardenburg syndrome	3.496733	0.038943	0.134993	NA
ENSDARG00000012397	*eya4*	*EYA4*	AD non-syndromic deafness	3.495119	0.000225	**0.001968**	Kozlowski et al., 2005; Wang et al., 2008
ENSDARG00000069423	*tmie*	*TMIE*	AR non-syndromic deafness	3.285445	0.00012	**0.001138**	Shen et al., 2008; Gleason et al., 2009
ENSDARG00000068166	*dfnb31b*	*WHRN*	Usher syndrome type 2D, AR non-syndromic deafness	2.864421	0.010032	**0.047095**	Blanco-Sánchez et al., 2014
ENSDARG00000011407	*col2a1b*	*COL2A1*	Stickler syndrome	2.802449	0.002457	**0.014951**	NA
ENSDARG00000102128	*eps8*	*EPS8*	AR non-syndromic deafness	2.782384	0.000132	**0.001236**	NA
ENSDARG00000010186	*myo3a*	*MYO3A*	AR non-syndromic deafness	2.523912	0.011106	0.051163	NA
ENSDARG00000045302	*smpx*	*SMPX*	X-linked non-syndromic deafness	2.435658	0.000595	**0.004516**	NA
ENSDARG00000042707	*cx30.3*	*GJB2*	AD keratitis-ichthyosis-deafness syndrome, AD and AR nonsyndromic deafness	2.33159	0.000102	**0.000991**	Tao et al., 2010; Chang-Chien et al., 2014
ENSDARG00000002831	*col4a4*	*COL4A4*	Alport syndrome	2.309518	0.032912	0.119212	NA
ENSDARG00000003395	*col4a3*	*COL4A3*	Alport syndrome	2.136137	0.03126	0.114457	NA
ENSDARG00000026165	*col11a1a*	*COL11A1*	Stickler syndrome	2.073293	0.025006	0.096541	Thisse and Thisse, 2004; Fang et al., 2010 (otic vesicle)

*AR, autosomal recessive; AD, autosomal dominant*.

### *Tg(myo6b:RiboTag)* gene expression profile is comparable to previously published FACS-based HC transcriptomes

**Figure 4:**
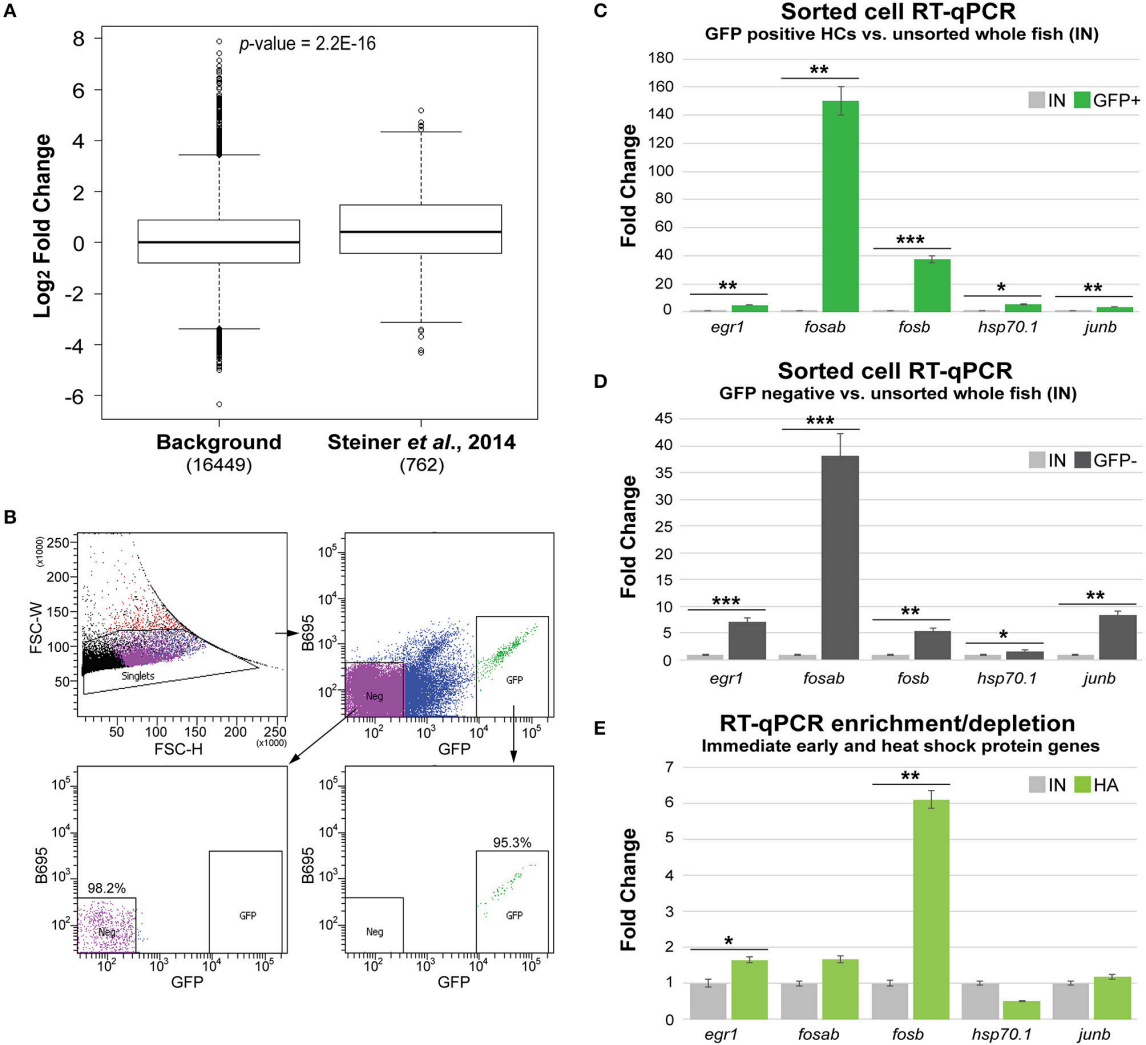
*Tg(myo6b:RiboTag)* immunoprecipitation avoids gene expression changes from cell sorting. **(A)** Sets of genes found to be enriched in sorted HCs from Steiner et al. (2014) have significantly higher fold change enrichment in the IP samples compared to remaining expressed genes (background). Significance was determined by Wilcoxon's test. **(B)** Cell sorting experiment utilizing the *Tg(myo6b:RiboTag)* model. Populations of both GFP positive (GFP+) and GFP negative (Neg, GFP–) were collected via FACS based on GFP expression. Post sort analysis shows high purity of negative and positive populations (98.2 and 95.3%, respectively). **(C–E)** RT-qPCR analysis of zebrafish homologs of immediate early and heat shock protein encoding genes in sorted HCs (GFP+, **C**), sorted non-HCs (GFP–, **D**), and immunoprecipitated RNA (IP, **C**) vs. RNA extracted from whole, non-dissociated larvae (IN). Error bars represent fold change ± standard deviation. Statistical significance was assessed by two-tailed Welch's *t*-test (*n* = 3). *p-value < 0.05, **p-value < 0.01, ***p-value < 0.001.

In order to ensure that similar HC gene enrichment could be obtained from our *Tg(myo6b:RiboTag)* immunoprecipitation method, we next wanted to compare our translatome dataset to a previously published cell sorting experiment. To do this, we utilized a dataset generated by comparing gene expression in sorted zebrafish HCs to sorted skin cells using microarray technology (Steiner et al., 2014). Setting a five-fold transcript enrichment criterion in HCs compared to skin cells within the microarray dataset, we identified 1,041 unique gene IDs with significant enrichment in sorted HCs (*p*-value < 0.05 from Steiner et al., 2014 results). We then analyzed the fold change values of this set of genes within our *Tg(myo6b:RiboTag)* dataset. Of the 1,041 genes identified as significantly enriched in the Steiner et al. dataset, 762 were identified as expressed in our dataset and could be used in this analysis. This is secondary to our strict CPM cutoff to determine gene expression (CPM > 0.01 for all IP and IN samples), as all 1,041 genes are found within our dataset before CPM filtering. Similar to the Steiner et al. dataset, the transcripts encoding these 762 genes also had a higher enrichment in HCs according to our dataset (IP vs. IN, mean Log_2_FC = 0.51), compared to all other transcripts detected as expressed (mean Log_2_FC = 0.085) (Figure 4A). This result suggests that similar HC expression results can be obtained using either cell sorting or the *Tg(myo6b:RiboTag)* zebrafish.

A possible concern with dissociation and FACS-based approaches for analysis of gene expression relates to induction of changes in gene expression secondary to cellular trauma, loss of tissue context (e.g., cell-cell contacts and lateral inhibition), and length of time from dissociation to RNA extraction. Indeed, a recent manuscript published by van den Brink et al. described robust induction of immediate early gene expression (i.e., *Fos, Jun*, and *Egr1*), as well as heat shock protein genes, in mouse muscle stem cells that were dissociated for single cell RNA-Seq (van den Brink et al., 2017). We therefore wanted to compare the expression of the zebrafish homologs of selected immediate early and heat shock protein encoding genes between sorted HCs and immunoprecipitated HC transcripts in the *Tg(myo6b:RiboTag)* IP. For this analysis, we utilized the concomitant *myo6b* promoter driven expression of GFP in the HCs of the *Tg(myo6b:RiboTag)*. Five-day-old larvae were dissociated and cells were separated by FACS to GFP-positive (HCs) and GFP-negative (cells from the rest of the fish) populations (Figure 4B). Using RT-qPCR, we found that the immediate early genes *egr1, fosab, fosb*, and *junb*, as well as the zebrafish heat shock protein gene *hsp70.1* were enriched in sorted HCs compared to their level of expression in intact fish (Figure 4C). Similarly, all five genes were highly expressed in the sorted GFP-negative cells in comparison to their expression in intact zebrafish (Figure 4D), suggesting that their expression is likely induced by the dissociation and cell sorting process. Finally, a similar RT-qPCR analysis performed on the *Tg(myo6b:RiboTag)* IP vs. IN samples showed that only two of these transcripts, *egr1* and *fosb* are enriched in the IP (Figure 4E). Taken together, these data suggest that (1) immediate early genes are induced in the sorted samples, and (2) that this technical artifact is avoided by measuring gene expression using the *Tg(myo6b:RiboTag)* zebrafish.

### Immunoprecipitation of the HC translatome reveals novel HC expressed genes

**Figure 5:**
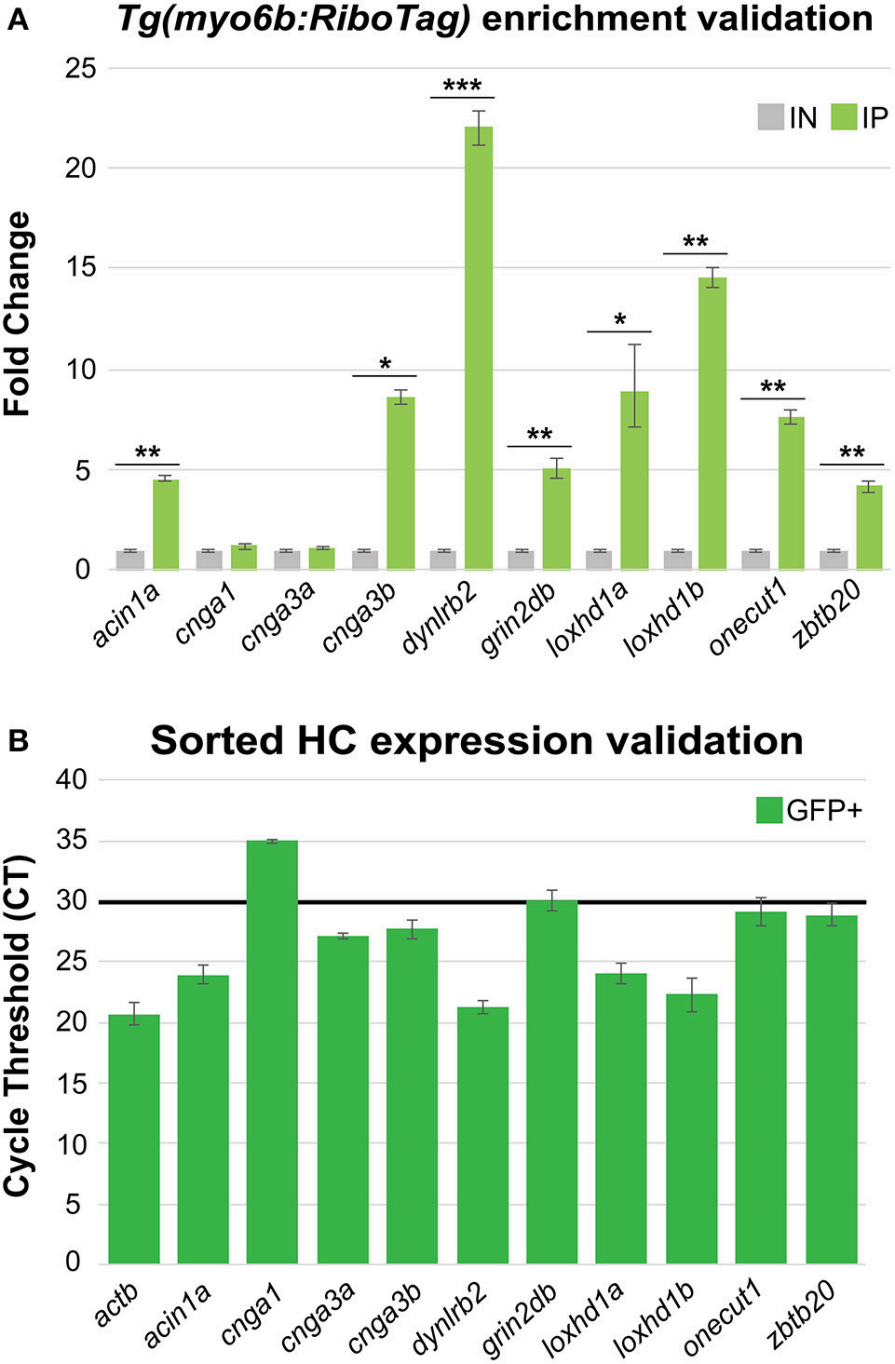
HC RiboTag immunoprecipitation reveals expression of novel HC expressed genes. **(A)** Validation of 10 genes found to be highly enriched in HCs from the *Tg(myo6b:RiboTag)* RNA-Seq using RT-qPCR in independent HC immunoprecipitation (IP) and input (IN) samples. Error bars represent fold change ± standard deviation, and statistical significance was assessed by two-tailed Welch's *t*-test (*n* = 3). *p-value < 0.05, **p-value < 0.01, ***p-value < 0.001. **(B)** Validation of the same 10 highly enriched genes selected from the *Tg(myo6b:RiboTag)* RNA-Seq in sorted HCs by RT-qPCR. Black bar indicates the cycle threshold (CT) cutoff used to denote reliable expression (CT ≤ 30). Error bars represent CT standard deviation between biological replicates (*n* = 3).

By comparing our data to genes previously identified as HC-specific in zebrafish, we have demonstrated that the *Tg(myo6b:RiboTag)* immunoprecipitation method allows for detection of HC expressed genes in zebrafish. However, many of the transcripts that were detected as highly enriched in the IP were not previously reported in the literature as expressed in zebrafish HCs. For this reason, we selected 10 genes that have not been previously characterized as expressed in zebrafish HCs from among our top 100 significantly enriched transcripts (see Supplementary Table 3) for validation of expression by RT-qPCR. These genes were *acin1a, cnga1, cnga3a, cnga3b, dynlrb2, grin2db, loxhd1a, loxhd1b, onecut1*, and *zbtb20*. First, we utilized independent *Tg(myo6b:RiboTag)* IP and IN samples, and found that eight out of the 10 genes validated as significantly enriched in IP compared to IN RNA samples (Figure 5A). To then further confirm the expression of these genes in HCs, we utilized RNA extracted from sorted HCs (post-sort purity = 95.3%, see Figure 4C). All 8 genes that validated as enriched in our IP vs. IN samples were also detected as expressed in sorted zebrafish HCs (cycle threshold [CT] values ≤ 30, Figure 5B). These data further show the utility of the *Tg(myo6b:RiboTag)* zebrafish in detecting HC expressed genes, as well as for the discovery of new genes with potential novel functions in HCs.

## Discussion

Cell type-specific expression analysis has gained popularity in the past decade, and is of particular importance to understanding the biology of rare cell types. The HCs of the zebrafish lateral line and inner ear are one such example of a rare cell type present within a complex tissue environment. The *Tg(myo6b:RiboTag)* zebrafish described here was generated to express both GFP and HA-tagged ribosomes in inner ear and lateral line HCs (Figures 1A-C). Thus, this model enables HC-specific gene expression analysis through two independent approaches: (1) tissue dissociation and cell sorting based on GFP expression, and (2) immunoprecipitation of HA-tagged ribosomes to enrich for HC-expressed transcripts. While HC-specific transcriptome analysis using flow cytometry is not novel, this is the first application of a RiboTag approach for immunoprecipitation of the zebrafish HC translatome, as well as the first model to allow HC-specific expression analysis using two separate approaches in zebrafish. Through the analyses presented in this manuscript, we have demonstrated the utility of the *Tg(myo6b:RiboTag)* model in enriching for HC-expressed transcripts (through the immunoprecipitation approach), while concurrently avoiding changes to gene expression that occur secondary to tissue dissociation and the cell sorting process. The immunoprecipitation protocol can be performed using either fresh or frozen tissues, making it a convenient technique for analysis of gene expression at multiple time points or treatment conditions, and removing the need for multiple cell sorting sessions. Our subsequent RNA-Seq and validation experiments revealed not only the expected enrichment of known zebrafish HC expressed transcripts, but also enrichment of transcripts with potential new functions in inner ear and/or lateral line HCs.

In light of these results, some important considerations should also be taken into account when using this model. First, unlike cell sorting where, in ideal conditions, the RNA will contain genetic material only from the cell types of interest, all ribosome immunoprecipitation models, including the *Tg(myo6b:RiboTag)* zebrafish, are not completely effective at isolating the translatome of a cell type of interest. Immunoprecipitated samples contain, to a varying extent, RNA from other cell types. Indeed, our average IP RNA yield (56 ng per 50 larvae at 5 dpf) was much larger than would be expected if this technique was specifically acquiring only actively translated RNA from HCs.

Second, the inclusion of transcripts from other cell types limits the interpretation of the data. The HC translatome results described in this manuscript were generated by comparing immunoprecipitated HC RNA to the whole fish transcriptome, detecting 2,379 genes as more than two-fold enriched in HCs. This method of comparison is not ideal for detecting all HC expressed transcripts, as it is estimated that an average 11,000–13,000 genes are expressed in all cell types (Ramsköld et al., 2009). Some transcripts which are expressed in HCs may not be detected as enriched if they are expressed in many other cell types included in the sample, as the effect of the depletion may (although not always), outweigh the effect of the enrichment. We therefore recommend that the *Tg(myo6b:RiboTag)* model be used to analyze gene expression in HCs between different conditions (e.g., drugs or noise) through direct comparison of the IP RNA. The IN RNA can be used to validate whether the changes in gene expression originate from HCs, and we recommend a two-fold enrichment cutoff in IP vs. IN to denote HC expression.

Third use of this model inherently relies on the expression of HA-tagged ribosomes in HCs under control of the *myo6b* promoter. This promoter is highly specific to HCs to all time points, which is critical to the successful use of this method and the purity of the immunoprecipitated RNA. However, analysis of the HC translatome in different conditions will therefore rely on (1) the presence of HCs and (2) the expression of *myo6b*. As examples, HC gene expression in very early developmental time points prior to the onset of *myo6b*, mutants in which HCs never develop or do not express *myo6b*, or treatments that rapidly decrease HC number or abolish *myo6b* expression, may be difficult or impossible to analyze with the *Tg(myo6b:RiboTag)* zebrafish.

Finally, the immunoprecipitation protocol is limited by the total amount of tissue that can be homogenized in one sample (we recommend not exceeding 10% the total volume of homogenization buffer). For analyses of the HC translatome at later developmental stages, different dissection techniques can be applied to reduce total tissue volume and “pre-enrich” for HCs (i.e., inner ear dissection, skin peeling, etc.). These dissections may also be applied to isolate and compare gene expression differences in specific HC subtypes (i.e., inner ear vs. neuromast). Overall, each of these points should be considered when designing experiments using this model.

Our results show that the *Tg(myo6b:RiboTag)* zebrafish immunoprecipitation protocol detailed in the methods yields quantities of RNA appropriate for downstream analyses of HC gene expression such as RT-qPCR and RNA-Seq. Additionally, this protocol is able to enrich for the zebrafish HC translatome, resulting in marked increases in transcript abundance of known HC expressed genes in the IP samples compared to RNA extracted from whole larvae (IN). Indeed, our RNA-Seq analysis of immunoprecipitated RNA shows enrichment for genes related to inner ear and lateral line HC function, while also showing depletion of genes involved in the function of other organs. Analysis of significantly enriched genes by RT-qPCR revealed high expression of genes not previously validated as expressed in zebrafish HCs, such as *loxhd1a, loxhd1b*, and *dynlrb2*, among others. *Loxhd1a* and *loxhd1b* are both homologs of the human deafness gene *LOXHD1*, the causative gene of DFNB77 (Grillet et al., 2009). Additionally, *dynlrb2*, a gene that encodes for a dynein light chain protein, has been previously identified as highly expressed in mouse HCs at both the transcript and protein levels, although its function in HCs has yet to be elucidated (Jiang et al., 2001; Scheffer et al., 2015; Shen et al., 2015; Hickox et al., 2017). Zebrafish may therefore be an appropriate model to study the roles that these genes play in normal HC function. Overall, taking into account the considerations outlined above, we believe that the *Tg(myo6b:RiboTag)* zebrafish represent an easy to use, versatile and sensitive model for studying inner ear and lateral line HC gene expression.

## Author contributions

AB and KK generated the model, MM, YO, KK, and RH designed and interpreted the experiments, MM, AB, YO, and NP performed the experiments, YS analyzed the gene expression data, and MM, KK, and RH wrote the manuscript.

### Conflict of interest statement

The authors declare that the research was conducted in the absence of any commercial or financial relationships that could be construed as a potential conflict of interest.

## References

[B1] Anders S. Huber W. (2010). Differential expression analysis for sequence count data. Genome Biol. 11:R106. 10.1186/gb-2010-11-10-r106 20979621 PMC3218662

[B2] Anders S. Pyl P. T. Huber W. (2015). Genome analysis HTSeq — a Python framework to work with high-throughput sequencing data. Bioinformatics 31, 166–169. 10.1093/bioinformatics/btu638 25260700 PMC4287950

[B3] Blanco-Sánchez B. Clément A. Fierro A. Jr. Washbourne P. Westerfield M. (2014). Complexes of Usher proteins preassemble at the endoplasmic reticulum and are required for trafficking and ER homeostasis. Dis. Models Mech. 7, 547–559. 10.1242/dmm.014068 24626987 PMC4007406

[B4] Burns J. C. Kelly M. C. Hoa M. Morell R. J. Kelley M. W. (2015). Single-cell RNA-Seq resolves cellular complexity in sensory organs from the neonatal inner ear. Nat. Commun. 6:8557. 10.1038/ncomms9557 26469390 PMC4634134

[B5] Chang-Chien J. Yen Y. C. Chien K. H. Li S. Y. Hsu T. C. Yang J. J.. (2014). The connexin 30.3 of zebrafish homologue of human connexin 26 may play similar role in the inner ear. Hear. Res. 313, 55–66. 10.1016/j.heares.2014.04.010 24811980

[B6] Chatterjee P. Padmanarayana M. Abdullah N. Holman C. L. LaDu J. Tanguay R. L.. (2015). Otoferlin deficiency in zebrafish results in defects in balance and hearing: rescue of the balance and hearing phenotype with full-length and truncated forms of mouse otoferlin. Mol. Cell. Biol. 35, 1043–1054. 10.1128/MCB.01439-14 25582200 PMC4333087

[B7] Di Donato V. Auer T. O. Duroure K. Del Bene F. (2013). Characterization of the calcium binding protein family in zebrafish. PLoS ONE 8:e53299. 10.1371/journal.pone.0053299 23341937 PMC3547026

[B8] Elkon R. Milon B. Morrison L. Shah M. Vijayakumar S. Racherla M.. (2015). RFX transcription factors are essential for hearing in mice. Nat. Commun. 6:8549. 10.1038/ncomms9549 26469318 PMC4634137

[B9] Erickson T. Nicolson T. (2015). Identification of sensory hair-cell transcripts by thiouracil-tagging in zebrafish. BMC Genomics 16:842. 10.1186/s12864-015-2072-5 26494580 PMC4619078

[B10] Erickson T. Morgan C. P. Olt J. Hardy K. Busch-Nentwich E. Maeda R. (2017). Integration of Tmc1/2 into the mechanotransduction complex in zebrafish hair cells is regulated by 3 Transmembrane O-methyltransferase (Tomt). Elife 5, 1–26. 10.7554/eLife.28474 PMC5462536 28534737

[B11] Fang M. Adams J. S. McMahan B. L. Brown R. J. Oxford J. T. (2010). The expression patterns of minor fibrillar collagens during development in zebrafish. Gene Exp. Patterns 10, 315–322. 10.1016/j.gep.2010.07.002 20647059 PMC2956583

[B12] Gay L. Karfilis K. V. Miller M. R. Doe C. Q. Stankunas K. (2014). Applying thiouracil tagging to mouse transcriptome analysis. Nat. Protoc. 9, 410–420. 10.1038/nprot.2014.023 24457332 PMC4112099

[B13] Gay L. Miller M. R. Ventura P. B. Devasthali V. Vue Z. Thompson H. L.. (2013). Mouse TU tagging: a chemical/genetic intersectional method for purifying cell type-specific nascent RNA. Genes Dev. 27, 98–115. 10.1101/gad.205278.112 23307870 PMC3553287

[B14] Gleason M. R. Nagiel A. Jamet S. Vologodskaia M. López-Schier H. Hudspeth A. J.. (2009). The transmembrane inner ear (Tmie) protein is essential for normal hearing and balance in the zebrafish. Proc. Natl. Acad. Sci. U.S.A. 106, 21347–21352. 10.1073/pnas.0911632106 19934034 PMC2781060

[B15] Grillet N. Schwander M. Hildebrand M. S. Sczaniecka A. Kolatkar A. Velasco J.. (2009). Mutations in LOXHD1, an evolutionarily conserved stereociliary protein, disrupt hair cell function in mice and cause progressive hearing loss in humans. Am. J. Hum. Genet. 85, 328–337. 10.1016/j.ajhg.2009.07.017. 19732867 PMC2771534

[B16] Harris M. Clark J. Ireland A. Lomax J. Ashburner M. Foulger R.. (2004). The Gene Ontology (GO) database and informatics resource. Nucleic Acids Res. 32, 258–261. 10.1093/nar/gkh036 14681407 PMC308770

[B17] Heiman M. Schaefer A. Gong S. Peterson J. Day M. Ramsey K. E. (2009). Development of a BACarray translational profiling approach for the molecular characterization of CNS cell types. Cell 14, 738–748. 10.1016/j.cell.2008.10.028 PMC2696821 19013281

[B18] Hertzano R. Elkon R. (2012). High throughput gene expression analysis of the inner ear. Hear. Res. 288, 77–88. 10.1016/j.heares.2012.01.002 22710153

[B19] Hickox A. E. Wong A. C. Y. Park K. Strojny C. Ramirez M. Yates J. R. (2017). Cellular/molecular global analysis of protein expression of inner ear hair cells, J. Neurosci. 37, 1320–1339. 10.1523/JNEUROSCI.2267-16.2016 28039372 PMC5296798

[B20] Howe D. G. Bradford Y. M. Conlin T. Eagle A. E. Fashena D. Frazer K.. (2013). ZFIN, the Zebrafish Model Organism Database : increased support for mutants and transgenics. Nucleic Acids Res. 41, 854–860. 10.1093/nar/gks938 23074187 PMC3531097

[B21] Jiang J. Yu L. Huang X. Chen X. Li D. Zhang Y.. (2001). Identification of two novel human dynein light chain genes, DNLC2A and DNLC2B, and their expression changes in hepatocellular carcinoma tissues from 68 Chinese patients. Gene 281, 103–113. 10.1016/S0378-1119(01)00787-9 11750132

[B22] Jiang L. Romero-Carvajal A. Haug J. S. Seidel C. W. Piotrowski T. (2014). Gene-expression analysis of hair cell regeneration in the zebrafish lateral line. Proc. Natl. Acad. Sci. U.S.A. 111, E1383–E1392. 10.1073/pnas.1402898111 24706903 PMC3986165

[B23] Kim D. Pertea G. Trapnell C. Pimentel H. Kelley R. Salzberg S. L. (2013). TopHat2: accurate alignment of transcriptomes in the presence of insertions, deletions and gene fusions. Genome Biol. 14:R36. 10.1186/gb-2013-14-4-r36 23618408 PMC4053844

[B24] Kindt K. S. Finch G. Nicolson T. (2012). Kinocilia mediate mechanosensitivity in developing zebrafish hair cells. Dev. Cell 23, 329–341. 10.1016/j.devcel.2012.05.022 22898777 PMC3426295

[B25] Kozlowski D. J. Whitfield T. T. Hukriede N. A. Lam W. K. Weinberg E. S. (2005). The zebrafish dog-eared mutation disrupts eya1, a gene required for cell survival and differentiation in the inner ear and lateral line. Dev. Biol. 227, 27–41. 10.1016/j.ydbio.2004.08.033 15572137

[B26] Kwan K. M. Fujimoto E. Grabher C. Mangum B. D. Hardy M. E. Campbell D. S.. (2007). The Tol2kit: a multisite gateway-based construction kit forTol2 transposon transgenesis constructs. Dev. Dyn. 236, 3088–3099. 10.1002/dvdy.21343 17937395

[B27] Lieschke G. J. Currie P. D. (2007). Animal models of human disease: zebrafish swim into view. Nat. Rev. 8, 353–367. 10.1038/nrg2091 17440532

[B28] Maeda R. Kindt K. S. Mo W. Morgan C. P. Erickson T. Zhao H.. (2014). Tip-link protein protocadherin 15 interacts with transmembrane channel-like proteins TMC1 and TMC2. Proc. Natl. Acad. Sci. U.S.A. 111, 12907–12912. 10.1073/pnas.1402152111 25114259 PMC4156717

[B29] Matern M. Vijayakumar S. Margulies Z. Milon B. Song Y. Elkon R.. (2017). Gfi1Cre mice have early onset progressive hearing loss and induce recombination in numerous inner ear non-hair cells. Sci. Rep. 7:42079. 10.1038/srep42079 28181545 PMC5299610

[B30] McDermott B. M. Baucom J. M. Hudspeth A. J. (2007). Analysis and functional evaluation of the hair-cell transcriptome. Proc. Natl. Acad. Sci. U.S.A. 104, 11820–11825. 10.1073/pnas.0704476104 17606911 PMC1905926

[B31] Millimaki B. B. Sweet E. M. Dhason M. S. Riley B. B. (2007). Zebrafish atoh1 genes: classic proneural activity in the inner ear and regulation by Fgf and Notch. Development 134, 295–305. 10.1242/dev.02734 17166920

[B32] Mudunuri U. Che A. Yi M. Stephens R. M. (2009). bioDBnet: the biological database network. Bioinformatics 25, 555–55610. 10.1093/bioinformatics/btn654 19129209 PMC2642638

[B33] Nicolson T. (2005). The genetics of hearing and balance in zebrafish. Ann. Rev. Genet. 39, 9–22. 10.1146/annurev.genet.39.073003.105049 16285850

[B34] Nicolson T. (2017). The genetics of hair-cell function in zebrafish. J. Neurogenet. 31, 102–112. 10.1080/01677063.2017.1342246 28705044 PMC6080859

[B35] Obholzer N. Wolfson S. Trapani J. G. Mo W. Nechiporuk A. Busch-Nentwich E. (2008). Cellular/molecular vesicular glutamate transporter 3 is required for synaptic transmission in zebrafish hair cells. J. Neurosci. 28, 2110–2118. 10.1523/JNEUROSCI.5230-07.2008 18305245 PMC6671858

[B36] Prykhozhij S. V. Marsico A. Meijsing S. H. (2013). Zebrafish Expression Ontology of Gene Sets (ZEOGS): a tool to analyze enrichment of zebrafish anatomical terms in large gene sets. Zebrafish 10, 303–315. 10.1089/zeb.2012.0865 23656298 PMC3760060

[B37] Ramsköld D. Wang E. T. Burge C. B. Sandberg R. (2009). An abundance of ubiquitously expressed genes revealed by tissue transcriptome sequence data. PLoS Comput. Biol. 5:e1000598. 10.1371/journal.pcbi.1000598 20011106 PMC2781110

[B38] Roh H. C. Tsai L. T.-Y. Lyubetskaya A. Tenen D. E. Kumari M. Rosen E.. (2017). Simultaneous transcriptional and epigenomic profiling from specific cell types within heterogeneous tissues *in vivo*. Cell Rep. 18, 1048–1061. 10.1016/j.celrep.2016.12.087 28122230 PMC5291126

[B39] Sanz E. Yang L. Su T. Morris D. R. McKnight G. S. Amieux P. S. (2009). Cell-type-specific isolation of ribosome-associated mRNA from complex tissues. Proc. Natl. Acad. Sci. U.S.A. 106, 13939–13944. 10.1073/pnas.0907143106 19666516 PMC2728999

[B40] Scheffer I. Shen J. Corey D. P. Chen Z. Y. (2015). Gene expression by mouse inner ear hair cells during development. J. Neurosci. 35, 6366–6380. 10.1523/JNEUROSCI.5126-14.2015 25904789 PMC4405555

[B41] Seiler C. Ben-David O. Sidi S. Hendrich O. Rusch A. Burnside B.. (2004). Myosin VI is required for structural integrity of the apical surface of sensory hair cells in zebrafish. Dev. Biol. 272, 328–338. 10.1016/j.ydbio.2004.05.004 15282151

[B42] Seiler C. Finger-Baier K. C. Rinner O. Makhankov Y. V. Schwarz H. Neuhauss S. C.. (2005). Duplicated genes with split functions: independent roles of protocadherin15 orthologues in zebrafish hearing and vision. Dev. Dis. 132, 615–623. 10.1242/dev.01591 15634702

[B43] Shen J. Scheffer D. I. Kwan K. Y. Corey D. P. (2015). SHIELD: an integrative gene expression database for inner ear research. Database 2015:bav071. 10.1093/database/bav071 26209310 PMC4513695

[B44] Shen Y.-C. Jeyabalan A. K. Wu K. L. Hunker K. L. Kohrman D. C. Thompson D. L.. (2008). The transmembrane inner ear (tmie) gene contributes to vestibular and lateral line development and function in the zebrafish (*Danio rerio*). Dev. Dyn. 237, 941–952. 10.1002/dvdy.21486 18330929 PMC3082740

[B45] Steiner A. B. Kim T. Cabot V. Hudspeth A. J. (2014). Dynamic gene expression by putative hair-cell progenitors during regeneration in the zebrafish lateral line. Proc. Natl. Acad. Sci. U.S.A. 111, E1393–E1401. 10.1073/pnas.1318692111 24706895 PMC3986164

[B46] Tao L. DeRosa A. M. White T. W. Valdimarsson G. (2010). Zebrafish cx30.3: identification and characterization of a gap junction gene highly expressed in the skin. Dev. Dyn. 239, 2627–2636. 10.1002/dvdy.22399 20737512 PMC2967642

[B47] Thisse B. Thisse C. (2004). Fast Release Clones: A High Throughput Expression Analysis., ZFIN Direct Data Submission.

[B48] Tryon R. C. Pisat N. Johnson S. L. Dougherty J. D. (2013). Development of translating ribosome affinity purification for zebrafish. Genesis 192, 187–192. 10.1002/dvg.22363 PMC3638809 23281262

[B49] Van Camp G. Smith R. (2017). Hereditary Hearing Loss Homepage. Available online at: http://hereditaryhearingloss.org

[B50] van den Brink S. C. Sage F. Vértesy Á. Spanjaard B. Peterson-Maduro J. Baron C. S.. (2017). Single-cell sequencing reveals dissociation-induced gene expression in tissue subpopulations. Nat. Methods 14, 935–936. 10.1038/nmeth.4437 28960196

[B51] Wang L. Sewell W. F. Kim S. D. Shin J. T. MacRae C. A. Zon L. I. (2008). Eya4 regulation of NA+/K+-ATPase is required for sensory development in zebrafish. Development 135, 3425–3434. 10.1242/dev.012237 18799547

