# Corrigendum: Transcriptomic Profiling of Zebrafish Hair Cells Using RiboTag

**DOI:** 10.3389/fcell.2018.00084

**Published:** 2018-08-27

**Authors:** Maggie S. Matern, Alisha Beirl, Yoko Ogawa, Yang Song, Nikhil Paladugu, Katie S. Kindt, Ronna Hertzano

**Affiliations:** ^1^Department of Otorhinolaryngology Head and Neck Surgery, University of Maryland School of Medicine, Baltimore, MD, United States; ^2^Section on Sensory Cell Development and Function, National Institute on Deafness and Other Communication Disorders, Bethesda, MD, United States; ^3^Institute for Genome Sciences, University of Maryland School of Medicine, Baltimore, MD, United States; ^4^Department of Anatomy and Neurobiology, University of Maryland School of Medicine, Baltimore, MD, United States

**Keywords:** inner ear, hair cells, zebrafish, RiboTag, RNA-Seq

Although the reported fold change values and statistics were accurate, there was a mistake in the CPM values shown in Supplementary Dataset 1 in the original article. This mistake has been corrected and it has resulted in a slight change in the number of transcripts meeting the CPM cutoff for expression (*n* = 17,164), enrichment (*n* = 2,379), and depletion (*n* = 2,258). These corrected numbers of expressed, enriched and depleted transcripts are now reflected in the text, as well as in Figure 3a. The gene ontology and ZEOGS analyses have been redone and changes have been made to Tables 1–3, and Supplementary Table 2. The genes chosen for validation now fall within the top 100 enriched transcripts rather than the top 50; therefore, Supplementary Table 3 has been changed to show the top 100 enriched transcripts. The scientific conclusions made from these analyses have not changed in any way.

**Figure 3:**
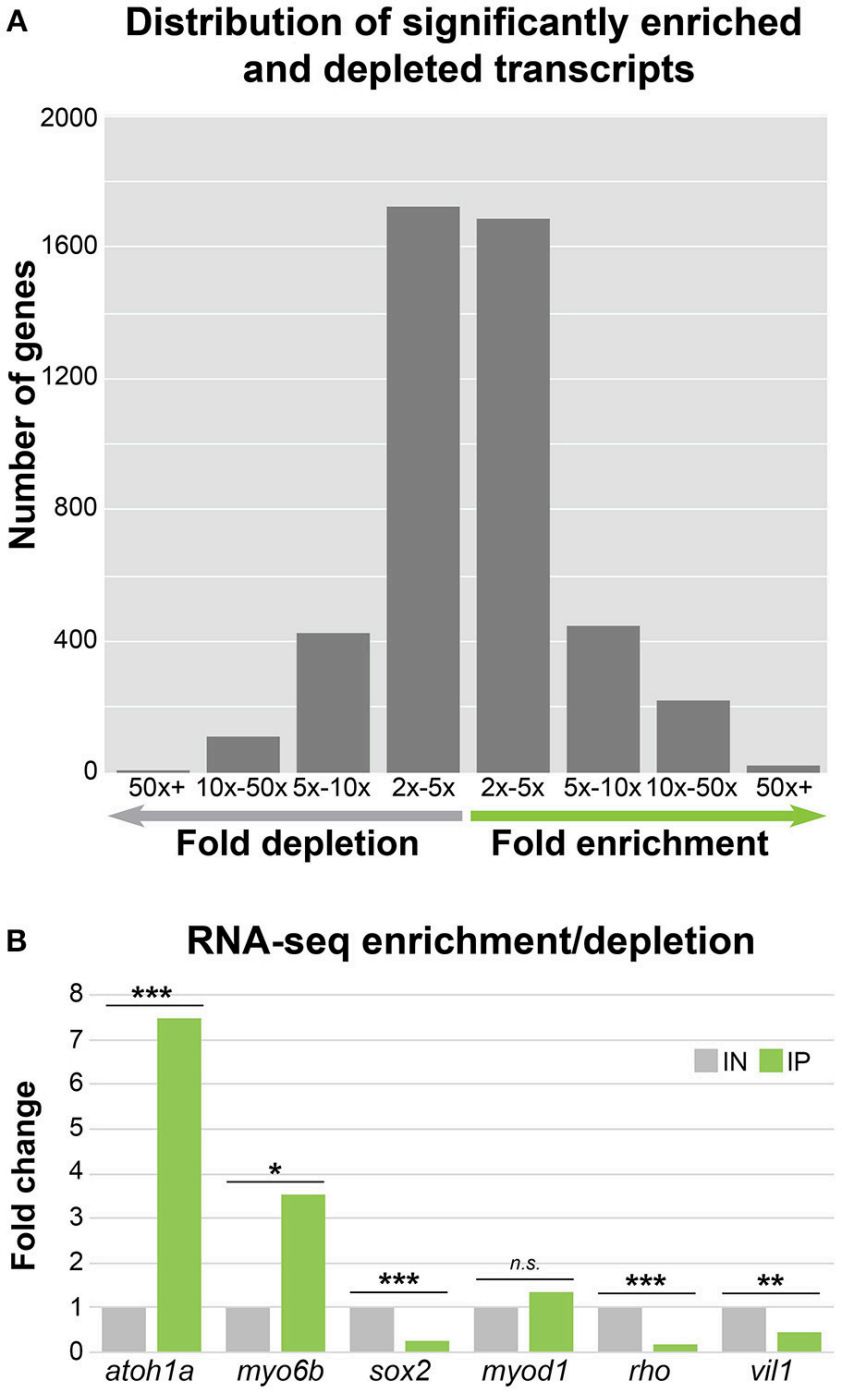
HC ribosome immunoprecipitation can reliably detect HC enriched and depleted transcripts by RNA-Seq. **(A)** Bar graph showing the distribution of significantly enriched and depleted transcripts in the IN vs. IP samples binned by fold change range. For genes with depleted transcripts in the IP samples, the number of genes per bin is as follows: 2x − 5x = 1726, 5x − 10x = 422, 5x − 10x = 109, and 50x + = 1. For IP enriched gene transcripts, the number of genes per bin is as follows: 2x − 5x = 1685, 5x − 10x = 450, 5x − 10x = 223, and 50x + = 21. **(B)** RNA-Seq fold change enrichment and depletion of HC expressed and non-expressed genes replicates the results obtained by RT-qPCR (Figure 2C). Statistical significance was assessed using DEseq (see Methods). *FDR < 0.05, **FDR < 0.01, ***FDR < 0.001.

**Table 1:** Gene ontology analysis of hair cell enriched genes.

**GO biological process complete**	**# in Reference**	**# in input**	**# expected**	**Fold enrichment**	**FDR**	**Genes**
miRNA mediated inhibition of translation	9	5	0.21	24.32	0.00252	*trim71 tnrc6b tnrc6a tnrc6c1 ago2*
Skeletal muscle contraction	23	5	0.53	9.52	0.0373	*CU929259 tnni1c tnnc1b CU929259 tnni1d*
**detection of mechanical stimulus**	25	5	0.57	8.76	0.0496	*tmc2a loxhd1a loxhd1b dennd5a lhfpl5a*
**sensory perception of mechanical stimulus**	48	7	1.1	6.37	0.0283	*tmc2a pcdh15b atp2b1a lhfpl5a mecp2 BX572619 dcdc2b*
**neuromast development**	62	8	1.42	5.64	0.0237	*atoh1a pcsk5a slc12a5b atp2b1a pho BX572619 erbb2 dcdc2b*
Steroid hormone mediated signaling pathway	77	9	1.76	5.11	0.0198	*vdra abhd2a pparda thrab rorb rorcb nr6a1b thrb nr0b2a*
mRNA metabolic process	299	19	6.84	2.78	0.0182	*celf4 ptbp3 qkib rbbp6 rbmx2 crnkl1 snrnp70 tnrc6a nova2 rbm25a exosc7 qkia kiaa0907 exosc3 coil snrpa aqr dicer1 ago2*
Transcription, DNA-templated	841	43	19.24	2.23	0.00093	*hdac4 hoxb1a pou6f2 vdra nfia rest stat2 hoxb3a foxh1 brf2 pparda hoxc6b bhlhe41 hoxa1a thrab rorb ncoa1 gtf2a1l nfat5a eed rorcb mef2b nrarpa mef2aa nr6a1b clocka med19a pou2f1b tcf3a jarid2b thrb onecut2 BX005395 twistnb onecut1 mecp2 ccnd1 hoxa1a znf367 nr0b2a pou2f2a asxl2 nfic*
Regulation of transcription, DNA-templated	1703	71	38.97	1.82	0.00092	*hdac4 hoxb1a nkap pou6f2 pde8b vdra nfia rest mkl2a stat2 hoxb3a foxh1 brf2 pparda hoxc6b has2 tcf3a bhlhe41 hoxa1a thrab gfi1aa trps1 rorb atoh1a foxj3 ncoa1 crebrf zfhx4 fosl1a nfat5a eed rfx7 rorcb tomm20a ches1 mef2b nrarpa CU633479 mef2aa nr6a1b clocka hmbox1b med19a pou2f1b tcf3a jarid2b thrb BX511021 cica onecut2 pbxip1b zfhx3 BX005395 onecut1 mecp2 rbpja foxb1b ccnd1 hnf1a hoxa1a tbl1xr1b mycbp znf367 nr0b2a pou2f2a crtc1a ago2 asxl2 nfic dot1l rbpjb*

**Table 2:** Gene ontology analysis of hair cell depleted genes.

**GO biological process complete**	**# in Reference**	**# in Input**	**# expected**	**Fold enrichment**	**FDR**	**Genes**
Antibiotic catabolic process	4	3	0.07	41.55	0.024	*esd cat amdhd2*
ATP synthesis coupled electron transport	43	7	0.78	9.02	0.00537	*AC024175.9 (*associated with *mt-nd4l mt-cyb mt-nd2 mt-co1 mt-nd4 mt-nd5)*
**Detection of light stimulus**	38	6	0.69	8.75	0.0176	*opn1mw1 gnat2 rho lamc1 opn1sw1 (*also associated with *opn1sw2)*
Pyruvate metabolic process	43	6	0.78	7.73	0.0275	*hkdc1 aldoca eno2 pdha1b aldoaa pkmb*
ATP biosynthetic process	64	8	1.16	6.93	0.00767	*hkdc1 aldoca eno2 AC024175.9 (*associated with *mt-atp6) aldoaa BX901937 pkmb*
**Visual perception**	97	12	1.75	6.85	0.00022	*opn1mw1 kera gnat2 rho lamc1 cryaa crx aoc2 irbp vsx2 opn1sw1 (*also associated with *opn1sw2)*
Proton transmembrane transport	57	7	1.03	6.8	0.0194	*atpv0e2 AC024175.9 (*associated with *mt-co1 mt-atp6) BX901937 atp6v0b atp6v1b2*
Regulation of cell growth	97	9	1.75	5.14	0.0179	*igfbp7 osgn1 ncaldb chrna1 casp9 epb41l3b lamtor2 dpysl2b arl3l1*
Coenzyme biosynthetic process	116	9	2.1	4.3	0.0436	*aldoca eno2 spra mat2al pdha1b aldoaa ndufa9b pkmb*
Nucleobase-containing compound catabolic process	153	11	2.77	3.98	0.0239	*hkdc1 aldoca eno2 upf3a smg5 pcid2 polr2gl aldoaa dis3 dnase1l3l pkmb*
Cellular protein localization	497	23	8.99	2.56	0.0115	*vps11 copb1 ap2m1b ap4e1 smg5 tomm40l vps29 pcid2 glrbb chrna1 atg9a copa wipi2 epb41l3b BX901937 grpel2 nmd3 hsc70 sx1b napgb pttg1ipb sec24d lamtor2*
Protein transport	530	24	9.59	2.51	0.0113	*arcn1b vps11 rab4a copb1 ap2m1b ap4e1 smg5 tomm40l vps29 pcid2 rab10 tsg101a atg9a copa tvp23b BX901937 (*associated with *Zgc:165520 vps37c) grpel2 nmd3 snx1b napgb jagn1a pttg1ipb sec24d*
System development	2,933	79	52.94	1.49	0.0437	*tfap2b cdh7 tyrp1b inpp5b wif1 vps11 rab4a ecrg4b igfbp7 copb1 slc2a2 slc35b2 col17a1b ponzr1 epb41b ncaldb fosab rab10 slc4a1a PPP1CC pou4f2 slc33a1 dacha ruvbl2 chrna1 snapc2 gdpd3a celsr2 copa rapsn ephb4a smyd1b casp9 lingo2a inpp5kb mcm3 olfm2a aldoaa grhl2a rtn4r BX470189 fez1 lamc1 dhps cryaa itm2bb id4 klhl40a camk1db crx lrfn4b rel lhx1a jagn1a inab scinla lamb2 padi2 anos1a slitrk6 zic6 CRIP2 plppr1 pttg1ipb sec24d polr3b ugdh SLITRK1 atp6v0b dpysl2b mab21l2 pou3f1 arl3l1 hpse lrrn1 PDCL3 dnase1l3l spry4 six3a*

**Table 3:** ZEOGS analysis of HC enriched transcripts.

**Anatomical term**	**Corrected *p*-value**	**Genes**
Neuromast	0.00052	*cabp2b morn3 pcsk5a rorb s100t atp2b1a gfi1aa otofa wasa pho bdnf pvalb8 tmc2a tmc2b s100s myclb atoh1a*
Levator operculi	0.06849	*tnnc1b smyhc2*
Hair cell anterior macula	0.07032	*atp2b1a tmc2a otofa*
Hyohyoideus	0.07515	*myha tnnc1b smyhc2*
Olfactory epithelium	0.0832	*tnks1bp1 cnga3a s100t dlg2 bdnf s100s s100a1 elavl3*
Olfactory bulb	0.0915	*klf7a mef2aa fabp10b plxnb2b s100t dlg2 bdnf pvalb8 s100s cadm1b igdcc3*
Hair cell posterior macula	0.09839	*atp2b1a tmc2a*

A fourth input sample, which was included in the original analysis, was missing from Supplementary Dataset 1 and Supplementary Table 1 in the original article. This sample has now been added to both the files. Sentence one in the subsection “RNA sequencing and informatics” under “Materials and Methods”, now reads “RNA from 5 dpf *Tg (myo6b:RiboTag)* zebrafish IN and IP samples was submitted in biological quadruplicates for RNA-Seq at the UMSOM Institute for Genome Sciences.” The following sentence has also been added: “One IP sample had a high intergenic content suggestive of DNA contamination and was excluded from the analysis.” Finally, sentence two in paragraph one of the subsection “RNA sequencing of IP and IN samples from *Tg(myo6b:RiboTag)* zebrafish” under “Results”, now reads “We therefore performed RNA-Seq on IP and IN samples in at least three biological replicates from 5 dpf *Tg(myo6b:RiboTag)* zebrafish”.

The authors apologize for these errors and state that this does not change the scientific conclusions of the article in any way.

The original article has been updated.

## Conflict of interest statement

The authors declare that the research was conducted in the absence of any commercial or financial relationships that could be construed as a potential conflict of interest.

