# Supplementary material for: Transcriptomic Profiling of Zebrafish Hair Cells Using RiboTag
Source: Front Cell Dev Biol. 2018 May 1;6:47. doi: 10.3389/fcell.2018.00047 (PMC5939014; doi:10.3389/fcell.2018.00047)
Supplement: Supplementary file 1 [file Table_1.DOCX]

| **Sample ID** | **Total Reads** | **Total Mapped Reads** | **% Mapped** | **% Properly Paired** | **Uniquely Mapped** | **% Exonic** | **% Intronic** | **% Intergenic** | **Total Features** | **Features w/ Coverage** | **Avg RPKM** |
| --- | --- | --- | --- | --- | --- | --- | --- | --- | --- | --- | --- |
| IN1 | 67501716 | 58249644 | 86.29 | 69.57 | 51646259 | 83.37 | 4.54 | 12.09 | 32266 | 27755 | 8.703 |
| IN2 | 98770890 | 84326437 | 85.38 | 67.69 | 79306031 | 88.38 | 3.09 | 8.53 | 32266 | 27967 | 6.635 |
| IN3 | 123359042 | 103916222 | 84.24 | 70.58 | 95564195 | 86.8 | 3.29 | 9.91 | 32266 | 27835 | 7.554 |
| IN4 | 119521966 | 102538913 | 85.79 | 69 | 96039668 | 88.34 | 2.95 | 8.71 | 32266 | 28092 | 6.873 |
| IP1 | 70148178 | 41564701 | 59.25 | 78.77 | 34889089 | 70.88 | 9.13 | 19.98 | 32266 | 25117 | 8.625 |
| IP2 | 60411404 | 35523712 | 58.8 | 80.86 | 26164121 | 59.41 | 11.49 | 29.1 | 32266 | 24817 | 12.976 |
| IP3 | 67794378 | 37278417 | 54.99 | 81.53 | 27142911 | 52.51 | 15.65 | 31.84 | 32266 | 24025 | 13.992 |

**Supplementary table 1. *Tg(myo6b:RiboTag)* RNA-Seq alignment statistics.** RNA-Seq alignment statistics of paired immunoprecipitation (IP) and input (IN) samples.
