# Supplementary material for: Transcriptomic Profiling of Zebrafish Hair Cells Using RiboTag
Source: Front Cell Dev Biol. 2018 May 1;6:47. doi: 10.3389/fcell.2018.00047 (PMC5939014; doi:10.3389/fcell.2018.00047)
Supplement: Supplementary file 2 [file Table_2.DOCX]

| **Anatomical term** | **corrected P-value** | **Genes** |
| --- | --- | --- |
| retinal photoreceptor layer | 0.0069 | *epb41l3b opn1sw1 irbp fam13a arl3l1 crx opn1mw1 scl35a3a gc3 gnat1 rho gnb5b gnat2 selt1a* |
| retina | 0.0107 | *nrip1a fez1 rtn4r tfap2b dacha epb41l3b anp32b opn1sw1 mab21l2 rrm2b sh3gl2 crhbp scinla gadd45gb.1 olfm2a pttg1ipb irbp vps11 fam13a ext1c arl3l1 slc6a1b vdac3 cat spry4 polr3b got1 id4 mt-nd2 crx hmgb1b scg3 rab4a lamc1 eno2 rgs8 tspan36 rogdi opn1mw1 slc25a3a gc3 slc29a1a phc2b tyrp1b tbr1b lrrn1 aldoaa zgc:91999 gnat1 rho gnb5b six3a dhcr7 gnb1a amdhd2 anxa11b kcnip3a gfap gnb3b sgce lbr gnat2 selt1a vsx2* |
| retinal pigmented epithelium | 0.0188 | *dct pttg1ipb irbp vps11 crx lamc1 tspan36 tyrp1b rho* |
| epidermis | 0.0247 | *epb41l3b padi2 caspb scinla cfl1l cfd aldh3b1 si:dkey-251i10.2 capn9 lamc1 rcn1 osgn1 alox12 kera atp1b1b wdr1 pycard cd151l gnb1a anxa11b lye plscr3b selt1a* |
| melanocyte | 0.0252 | *spra dct tyr tspan36 tyrp1b* |
| ocular blood vessel | 0.0257 | *igfbp7 gfap* |
| epiphysis | 0.0498 | *fez1 inab ephb4a lrrn3 sh3gl2 necap1 map1lc3b irbp arl3l1 qsox1 crispld1b crx scg3 dpysl2b igfbp7 rogdi opn1mw1 atp1b1b slc25a3a lrrn1 atpv0e2 gnat1 rho irf2bp2a gnb5b gnb3a gnat2* |
| peripheral olfactory organ | 0.0559 | *inab ppp1r14ba epb41l3b acy1 padi2 scinla cyp3c1 aldh3b1 fam13a capn9 serpine2 ftr83 alox12 atp1b1b wdr1 krt18 spock2 stk38l tbr1b pycard aldoaa irf2bp2a gnb1a anxa11b fosab selt1a* |
| corneal epithelium | 0.0824 | *scinla kera* |

**Supplementary table 2. ZEOGS analysis of HC depleted transcripts.** Detection of overrepresented anatomical structures in the *Tg(myo5b:RiboTag)* RNA-Seq highly depleted gene set (five-fold or greater depletion in the IP compared to IN samples, n=532).
