# Supplementary material for: Transcriptomic Profiling of Zebrafish Hair Cells Using RiboTag
Source: Front Cell Dev Biol. 2018 May 1;6:47. doi: 10.3389/fcell.2018.00047 (PMC5939014; doi:10.3389/fcell.2018.00047)
Supplement: Supplementary file 3 [file Table_3.DOCX]

| **Ensembl ID** | **Gene name** | **IP/IN FC** | **Ensembl ID** | **Gene name** | **IP/IN FC** |
| --- | --- | --- | --- | --- | --- |
| ENSDARG00000070620 | ***grin2db*** | 238.45073 | ENSDARG00000097891 | *si:ch73-4e5.1* | 29.65151 |
| ENSDARG00000012297 | ***cnga3b*** | 171.14503 | ENSDARG00000015543 | *s100a1* | 29.46444 |
| ENSDARG00000095475 | *si:ch211-196j1.2p* | 140.54253 | ENSDARG00000101240 | *CU651662.2* | 29.20299 |
| ENSDARG00000070726 | ***cnga3a*** | 120.51259 | ENSDARG00000098668 | *si:dkeyp-7b3.2* | 28.98217 |
| ENSDARG00000005586 | ***zbtb20*** | 109.13792 | ENSDARG00000086048 | *si:ch211-229i14.2* | 28.90909 |
| ENSDARG00000103864 | *CABZ01117436.1* | 108.30144 | ENSDARG00000057276 | *iqca1* | 27.31372 |
| ENSDARG00000025797 | *abhd2a* | 105.04717 | ENSDARG00000083557 | *dre-mir-125b-3* | 27.17617 |
| ENSDARG00000093232 | *si:dkey-202p8.1* | 98.87874 | ENSDARG00000041301 | *crybb3* | 27.09172 |
| ENSDARG00000007982 | ***onecut1*** | 87.784253 | ENSDARG00000020845 | *tns1b* | 27.00964 |
| ENSDARG00000033104 | *tmc2a* | 85.964033 | ENSDARG00000090387 | *onecut2* | 26.96214 |
| ENSDARG00000056929 | *kdm6bb* | 83.536804 | ENSDARG00000077360 | *zgc:173593* | 26.08159 |
| ENSDARG00000093487 | *si:dkey-217f16.2* | 79.719269 | ENSDARG00000078832 | *si:dkey-73p2.3* | 25.14349 |
| ENSDARG00000074638 | ***loxhd1b*** | 77.354843 | ENSDARG00000054290 | ***acin1a*** | 25.11392 |
| ENSDARG00000036844 | *tsen54* | 76.456421 | ENSDARG00000007245 | *rundc3aa* | 24.93577 |
| ENSDARG00000044212 | *CR735126.1* | 67.599114 | ENSDARG00000011602 | *si:dkeyp-117h8.2* | 24.34544 |
| ENSDARG00000068760 | *tnks1bp1* | 65.117677 | ENSDARG00000095639 | *si:dkey-177p2.5* | 24.33792 |
| ENSDARG00000087953 | *wu:fi09b08* | 55.900177 | ENSDARG00000092945 | *si:ch211-250g4.3* | 24.31019 |
| ENSDARG00000096831 | *si:dkey-73p2.5* | 55.218546 | ENSDARG00000073936 | *BX511021.2* | 24.06788 |
| ENSDARG00000061268 | *ago2* | 53.500537 | ENSDARG00000014209 | *ODF3L2 (1 of many)* | 23.56497 |
| ENSDARG00000036462 | *rab11fip1b* | 50.795805 | ENSDARG00000095906 | *si:dkey-235h8.1* | 23.25235 |
| ENSDARG00000089602 | *si:dkey-217f16.1* | 50.01916 | ENSDARG00000043757 | *ptbp3* | 22.35869 |
| ENSDARG00000094738 | ***loxhd1a*** | 47.780062 | ENSDARG00000056917 | *si:rp71-45g20.4* | 22.31696 |
| ENSDARG00000101205 | *si:dkey-242k1.6* | 46.817407 | ENSDARG00000096967 | *si:dkey-207j16.10* | 21.72401 |
| ENSDARG00000099968 | *BX322612.1* | 45.599014 | ENSDARG00000070440 | *atp6v1c2* | 21.68779 |
| ENSDARG00000101455 | *si:ch211-284k5.2* | 45.304755 | ENSDARG00000078775 | *RASA2* | 21.6853 |
| ENSDARG00000075972 | *csrnp2* | 44.36362 | ENSDARG00000099178 | *CABZ01033309.1* | 21.40067 |
| ENSDARG00000012125 | ***cnga1*** | 43.513005 | ENSDARG00000003998 | *phyhipla* | 21.31558 |
| ENSDARG00000087429 | *si:dkey-73p2.2* | 43.238953 | ENSDARG00000056617 | *rpgra* | 21.19264 |
| ENSDARG00000102275 | *CU104716.1* | 43.234123 | ENSDARG00000105114 | *crebrf* | 20.96328 |
| ENSDARG00000079688 | *tnrc6a* | 41.767079 | ENSDARG00000077741 | *zgc:175135* | 20.56711 |
| ENSDARG00000052294 | *BX537277.1* | 41.605431 | ENSDARG00000031756 | *mef2aa* | 20.5153 |
| ENSDARG00000087337 | *wu:fi09b08* | 40.840477 | ENSDARG00000030311 | *tmc2b* | 20.28182 |
| ENSDARG00000090886 | *CU856539.4* | 39.586324 | ENSDARG00000018004 | *nkx2.5* | 20.25081 |
| ENSDARG00000089952 | ***DYNLRB2*** | 39.354195 | ENSDARG00000035133 | *pho* | 20.13909 |
| ENSDARG00000086573 | *omga* | 38.739876 | ENSDARG00000028213 | *ttn.2* | 19.90842 |
| ENSDARG00000099005 | *palm2* | 38.006009 | ENSDARG00000075542 | *zfhx4* | 19.90344 |
| ENSDARG00000102437 | *BX321875.2* | 37.562198 | ENSDARG00000067509 | *slc24a4b* | 19.85265 |
| ENSDARG00000073857 | *klf7a* | 36.246227 | ENSDARG00000098702 | *zgc:165603* | 19.62371 |
| ENSDARG00000071727 | *si:dkey-37o8.1* | 35.866122 | ENSDARG00000069499 | *spag1b* | 19.59808 |
| ENSDARG00000093029 | *si:dkey-175d9.2* | 34.396799 | ENSDARG00000026406 | *anxa5a* | 19.5324 |
| ENSDARG00000090015 | *BX321875.1* | 33.741061 | ENSDARG00000070698 | *kbtbd8* | 19.42143 |
| ENSDARG00000105587 | *si:dkey-111f13.3* | 32.635428 | ENSDARG00000033450 | *nkap* | 19.20547 |
| ENSDARG00000015425 | *slc24a4a* | 32.455301 | ENSDARG00000076005 | *piezo2a.2* | 19.10798 |
| ENSDARG00000086059 | *CABZ01067746.1* | 31.974666 | ENSDARG00000071585 | *si:dkeyp-110e4.11* | 18.80712 |
| ENSDARG00000076847 | *tnrc6c1* | 31.966324 | ENSDARG00000006527 | *brd3a* | 18.73924 |
| ENSDARG00000077721 | *knop1* | 31.441963 | ENSDARG00000013863 | *fam133b* | 18.69995 |
| ENSDARG00000099283 | *epb41a* | 30.853552 | ENSDARG00000006923 | *cacna1ab* | 18.68518 |
| ENSDARG00000042677 | *cadm1b* | 30.441341 | ENSDARG00000036826 | *ankrd52a* | 18.40239 |
| ENSDARG00000095705 | *CDPF1* | 30.081108 | ENSDARG00000063730 | *osbpl6* | 18.37327 |
| ENSDARG00000078072 | *si:ch211-232i5.1* | 29.804177 | ENSDARG00000089110 | *si:ch211-198b3.4* | 18.3509 |

**Supplementary table 3. Top 100 significantly HC enriched transcripts.** *Tg(myo6b:RiboTag)* RNA-Seq results of the top 100 significantly enriched genes in the IP compared to IN samples (fold change > 2, IP CPM > 1, false discovery rate [FDR] < 0.05). Genes selected for validation by RT-qPCR are shown in bold.
