# Supplementary material for: Transcriptomic Profiling of Zebrafish Hair Cells Using RiboTag
Source: Front Cell Dev Biol. 2018 May 1;6:47. doi: 10.3389/fcell.2018.00047 (PMC5939014; doi:10.3389/fcell.2018.00047)
Supplement: Supplementary file 4 [file Table_4.DOCX]

***Supplementary material***

**Transcriptomic profiling of zebrafish hair cells using RiboTag**

**Maggie S. Matern, Alisha Beirl, Yoko Ogawa, Yang Song, Nikhil Paladugu, Katie Kindt, Ronna Hertzano^*^**

**Correspondence:**

Ronna Hertzano, M.D., Ph.D.

Department of Otorhinolaryngology Head and Neck Surgery

16 S Eutaw St. Suite 500, Baltimore, MD 21201

Phone: 410-328- 1892

Email address: [rhertzano@som.umaryland.edu](mailto:rhertzano@som.umaryland.edu)

| **Primer name** | **Sequence** | **Primer name** | **Sequence** |
| --- | --- | --- | --- |
| *atoh1a* forward | 5’-caacgccctgtccgacttac-3’ | *dynlrb2* forward | 5’-aaagaggattcaggcccaac-3’ |
| *atoh1a* reverse | 5’-gggagatcggtccgtttctaa-3’ | *dynlrb2* reverse | 5’-tgtccagcgtcgttctaatg-3’ |
| *myo6b* forward | 5’-ttggttcttcacagagatgggt-3’ | *grin2db* forward | 5’-tcttcctggccagctacact-3’ |
| *myo6b* reverse | 5’-ggtcgtacttcttcacccca-3’ | *grin2db* reverse | 5’-ctctgtggggtgctgaaact-3’ |
| *sox2* forward | 5’-actccatgaccaactcgcag-3’ | *loxhd1a* forward | 5’-gcaggtctacggagaaaacg-3’ |
| *sox2* reverse | 5’-atggagcccagtgtcattcc-3’ | *loxhd1a* reverse | 5’-ttcccgatatcaggcatctc-3’ |
| *myod1* forward | 5’-tcaatgacacaccaaatgctgac-3’ | *loxhd1b* forward | 5’-acggacaactttgagcaggg-3’ |
| *myod1* reverse | 5’-ccacgatgctggacagacaa-3’ | *loxhd1b* reverse | 5’-tgccagatacgcagtttggt-3’ |
| *rho* forward | 5’-aacctggagggcttctttgc-3’ | *onecut1* forward | 5’-tttcagagaatgtccgcgct-3’ |
| *rho* reverse | 5’-gaaacggaagttgctgaccg-3’ | *onecut1* reverse | 5’-cttggagatgtttccccgct-3’ |
| *vil1* forward | 5’-attgctccggtggcaggtat-3’ | *zbtb20* forward | 5’-gcgtgcactctttgctgtaa-3’ |
| *vil1* reverse | 5’-tctgaagtcctggtgtggttt-3’ | *zbtb20* reverse | 5’-aatccttcagcgagaacgaa-3’ |
| *actb1* forward | 5’-accatcggcaatgagcgttt-3’ | *egr1* forward | 5’-ggggagcagtttgatcacctt3’ |
| *actb1* reverse | 5’-ccgcagattccatacccagg-3’ | *egr1* reverse | 5’-ccgttgggtggagtaggtct-3’ |
| *acin1a* forward | 5’-cctcgtggcaagctgtcta-3’ | *fosab* forward | 5’-tacccgctcaaccagactca-3’ |
| *acin1a* reverse | 5’-tacagttcctgtgcggttca-3’ | *fosab* reverse | 5’-tggaccatccactgcaagtc-3’ |
| *cnga1* forward | 5’-ttatcggatgcggtgtacct-3’ | *fosb* forward | 5’-tctcagtacctgtcgtccgt-3’ |
| *cnga1* reverse | 5’-cgcgaagctttttctgatct-3’ | *fosb* reverse | 5’-ccagtggctgttacacactc-3’ |
| *cnga3a* forward | 5’-gctatcagttcgggcctctg-3’ | *hsp70*.1 forward | 5’-ccgacgaggtgttaattcgc-3’ |
| *cnga3a* reverse | 5’-gtcagaggagttgtcgtcaca-3’ | *hsp70*.1 reverse | 5’-cacacaggagtaggtggtgc-3’ |
| *cnga3b* forward | 5’-tgacacatttgtgagatcaaggac-3’ | *junba* forward | 5’-atgatactgaatgcgcgacg-3’ |
| *cnga3b* reverse | 5’-agtgctcccacagcttcttg-3’ | *junba* reverse | 5’-ctcgacagctgacgtccaaa-3’ |

**Supplementary table 4. RT-qPCR primers.** All primer sequences used for enrichment/depletion analyses, RNA-Seq validation and immediate early and heat shock protein encoding gene expression analyses.
